# BCG immunization mitigates SARS-CoV-2 replication in macaques via monocyte efferocytosis and neutrophil recruitment in lungs

**DOI:** 10.1172/jci.insight.194633

**Published:** 2025-08-08

**Authors:** Mohammad Arif Rahman, Katherine C. Goldfarbmuren, Sarkis Sarkis, Massimiliano Bissa, Anna Gutowska, Luca Schifanella, Ramona Moles, Melvin N. Doster, Hanne Andersen, Yogita Jethmalani, Leonid Serebryannyy, Timothy Cardozo, Mark G. Lewis, Genoveffa Franchini

**Affiliations:** 1 Animal Models and Retroviral Vaccines Section, Basic Research Laboratory, Center for Cancer Research (CCR), National Cancer Institute (NCI), National Institutes of Health (NIH), Bethesda, Maryland, USA.; 2 Advanced Biomedical Computational Science, Frederick National Laboratory for Cancer Research, Frederick, Maryland, USA.; 3 CCR Collaborative Bioinformatics Resource, NCI, NIH, Bethesda, Maryland, USA.; 4 Bioqual, Rockville, Maryland, USA.; 5 Vaccine Research Center, National Institute of Allergy and Infectious Diseases (NIAID), NIH, Bethesda, Maryland, USA.; 6 New York University Grossman School of Medicine, NYU Langone Health, New York, New York, USA.

**Keywords:** Immunology, Infectious disease, COVID-19, Innate immunity, Vaccines

## Abstract

Exposure to Bacillus Calmette-Guérin (BCG) or Canarypox ALVAC/Alum vaccine elicits pro- or antiinflammatory innate responses, respectively. We tested whether prior exposure of macaques to these immunogens protected against SARS-CoV-2 replication in lungs and found more efficient replication control after the pro-inflammatory immunity elicited by BCG. The decreased virus level in lungs was linked to early infiltrates of classical monocytes producing IL-8 with systemic neutrophils, Th2 cells, and Ki67^+^CD95^+^CD4^+^ T cells producing CCR7. At the time of SARS-CoV-2 exposure, BCG-treated animals had higher frequencies of lung infiltrating neutrophils and higher CD14^+^ cells expressing efferocytosis marker MERTK, responses correlating with decreased SARS-CoV-2 replication in lung. At the same time point, plasma IL-18, TNF-α, TNFSF-10, and VEGFA levels were also higher in the BCG group and correlated with decreased virus replication. Finally, after SARS-CoV-2 exposure, decreased virus replication correlated with neutrophils producing IL-10 and CCR7 preferentially recruited to the lungs of BCG-vaccinated animals. These data point to the importance of the spatiotemporal distribution of functional monocytes and neutrophils in controlling SARS-CoV-2 levels and suggest a central role of monocyte efferocytosis in curbing replication.

## Abstract 2

BCG vaccination promotes efferocytosis and other pro-inflammatory innate immune responses, contributing to the control of SARS-CoV-2 replication in the lungs of male macaques.

## Introduction

The outbreak of the COVID-19 disease, caused by SARS-CoV-2, originated in the Wuhan province of China in December 2019. By the spring of 2024, more than 700 million people had been infected with SARS-CoV-2 (compiled data mostly excluding asymptomatic and mild infections), with more than 7 million deaths caused by the disease. In the United States, more than 110 million people were infected, with more than 1.4 million dead (1). COVID-19 vaccines developed to induce adaptive immunity (2, 3) made great strides in preventing death but not in preventing the spread of ensuing virus variants. While adaptive responses such as antibodies and T cells clearly contribute to protection from COVID-19, the role of innate immune responses, particularly that of myeloid cells and neutrophils, in protective immunity is not completely understood (4–6).

Bacterial and viral vector-based vaccines elicit strong innate responses (7–11). Bacillus Calmette-Guérin (BCG) immunization, originally developed against *Mycobacterium tuberculosis* (12), induces pro-inflammatory trained immunity linked to IL-6, IL-8, and TNF-α expression by inducing epigenetic changes in innate immune memory responses in monocytes (13), neutrophils (14), and natural killer (NK) cells (15). BCG has been used to partially protect against TB for the past 100 years (16), as well as against unrelated infections (17–19). In newborn children, for example, BCG vaccination mitigates respiratory tract infections by other pathogens (17, 20), and in the murine model, BCG has also shown to be beneficial against fungal parasites and viral infections (21–23). Clinical data have suggested that BCG vaccination is associated with reduction of influenza symptoms in the elderly (24). In the context of the SARS-CoV-2 pandemic, epidemiological data in Bangladesh, India, the Philippines, Thailand, and Nepal, where the rate of BCG vaccination is high, the mortality rate due to COVID-19 infection has been relatively low (4–6, 25), suggesting that BCG vaccination may also mitigate SARS-CoV-2 infection.

The avian Canarypox ALVAC specifically infects CD14^+^ monocytes and dendritic cells (26, 27), transiently increases monocyte numbers, activates the inflammasome (28), and induces both pro-inflammatory cytokines, such as IL-2, IL-1β, IL-5, IL-6, MIP-1α, MIP-1β, MCP, VEGF, and IP-10, and antiinflammatory cytokines, such as IL-1Ra, IL-6 (which also acts as a pro-inflammatory cytokine), and IL-10 (26, 29–31). However, when ALVAC is delivered with the Alum adjuvant, an antiinflammatory immunological landscape prevails because of the recruitment of Tregs and augmented IL-10 production (28). ALVAC/Alum promotes trained immunity by metabolic and epigenetic reprogramming of CD14^+^ monocytes toward an M2-like phenotype (28). In addition, ALVAC/Alum-based vaccine regimens generate robust neutrophil responses (32). Finally, ALVAC/Alum-based vaccines induce mucosal NK cells/innate lymphoid cells (ILCs) activation (9, 28, 33) and generate memory NK cells (9), which play a role in preventing SIV/SHIV infection.

In the current study, we investigated the effect of pro-inflammatory M1-like monocytes and antiinflammatory M2-like monocytes, respectively induced by BCG or ALVAC/Alum vaccination, on SARS-CoV-2 infection and replication. Neither BCG nor ALVAC/Alum vaccination protected against SARS-CoV-2 infection as expected. However, prior BCG exposure was associated with more efficient control of SARS-CoV-2 replication in the lung by day 7 postinfection. BCG vaccination induces IL-8^+^ classical monocytes in blood and bronchoalveolar lavage (BAL), blood IL-8^+^ nonclassical monocytes, TNF-α^+^ intermediate monocytes, CCR7^+^ neutrophils, Th2 cells, and Ki67^+^CD95^+^CD4^+^ cells, while depleting neutrophils expressing CD162, CD11b, and myeloperoxidase (MPO). Prior to SARS-CoV-2 exposure (Pre-S), absolute neutrophil count, CD162^+^ neutrophils, IL-8^+^ classical monocytes, MERTK^+^ monocytes, IL-18, TNF-α, TNFSF-10, and VEGFA were higher in the blood of the BCG group compared with ALVAC/Alum. Following infection by SARS-CoV-2, CCR7^+^ neutrophils and IL-10^+^ neutrophils were higher and MPO^+^ neutrophils lower in the lungs of BCG-vaccinated animals. All of these responses in BCG animals were associated with decreased virus levels in the BAL. Taken together, these data suggest that prior exposure to BCG generates innate immune responses beneficial for the control of SARS-CoV-2 viremia.

## Results

### Lower SARS-CoV-2 replication in lung of macaques vaccinated with BCG compared with ALVAC/Alum.

**Figure 1:**
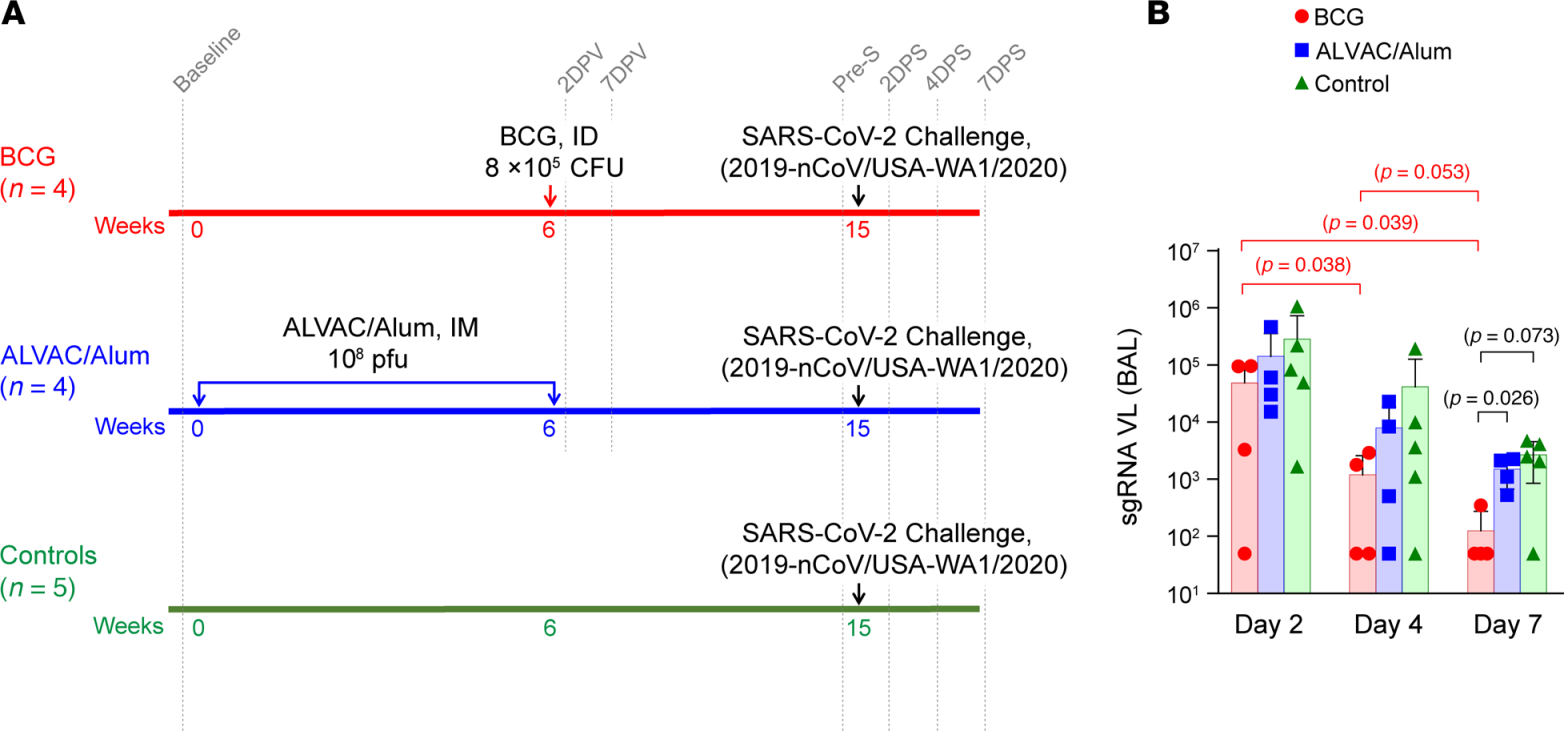
Schematic representation of the immunization regimen and replicating viral load in BAL. (**A**) Thirteen rhesus macaques included in this study were divided into 3 groups: BCG vaccine (*n* = 4), ALVAC/Alum vaccine (*n* = 4), and controls (*n* = 5). BCG vaccine was administered once intradermally (ID), and ALVAC/Alum vaccine was administered twice, 6 weeks apart, intramuscularly (IM). Controls remained untreated until challenge. Nine weeks after last immunization all the animals were exposed to 2019-nCoV/USA-WA1/2020 SARS-CoV-2 virus intranasally and intratracheally. DPV, days postvaccination; DPS, days post–SARS-CoV-2. (**B**) Replicating viral load (VL) in BAL post–SARS-CoV-2 viral challenge. Bar plots denote mean and error bars are SD. Red, blue, and green represent BCG, ALVAC/alum, and control, respectively. Statistical differences between groups for each time point were calculated by the Mann-Whitney/Wilcoxon test, and raw *P* values < 0.1 are displayed in black. Statistical differences between time points for each group separately were calculated by fitting generalized estimating equations with animal as a random effect, and raw *P* values < 0.1 are displayed in the color for the group tested. *P* values above 0.1 were omitted from the figure for clarity and can be found with multiple comparisons adjustments in Supplemental Figure 1F. sgRNA, subgenomic RNA.

We divided 13 macaques into 3 groups: 4 animals received a single dose of BCG; 4 animals received 2 doses of ALVAC/Alum vaccine; and a control group of 5 animals was left untreated (Figure 1A). Nine weeks following the completion of all vaccine regimens, the groups were exposed to SARS-CoV-2 via both intranasal and intratracheal routes. Nasal swabs, oropharyngeal swabs, and BAL fluid (the latter used as a surrogate of lung tissue) were collected on days 2, 4, and 7 after exposure to quantify SARS-CoV-2 viral load (VL), as these methods offer a less invasive alternative to tissue sampling. Primers targeting the nucleocapsid gene were used to detect viral input (VL), while primers targeting the subgenomic envelope (sgRNA) were used to assess viral replication (34). VL tended to decrease over time, and by day 7 BAL VL trended lower in the BCG group compared with the ALVAC/Alum-immunized animals (Supplemental Figure 1; supplemental material available online with this article; https://doi.org/10.1172/jci.insight.194633DS1). Accordingly, 3 out of 4 BCG-vaccinated animals were able to control SARS-CoV-2 replication in lungs by day 7 (Figure 1B). In contrast, only 1 animal from the unvaccinated group showed similar control of SARS-CoV-2 replication. None of the other animals, including those in the ALVAC/Alum-immunized group, demonstrated effective control of viral replication in the lung (Figure 1B). At the same time point, the level of replicating virus in the nasal and oropharyngeal compartments did not differ among the groups (Supplemental Figure 1). Taken together, these data suggest that prior vaccination with BCG is related to more efficient control of SARS-CoV-2 replication in the lung.

### Vaccine-associated monocyte IL-8 production exhibits opposite effect of IL-10 for SARS-CoV-2 replication in lung.

**Figure 2:**
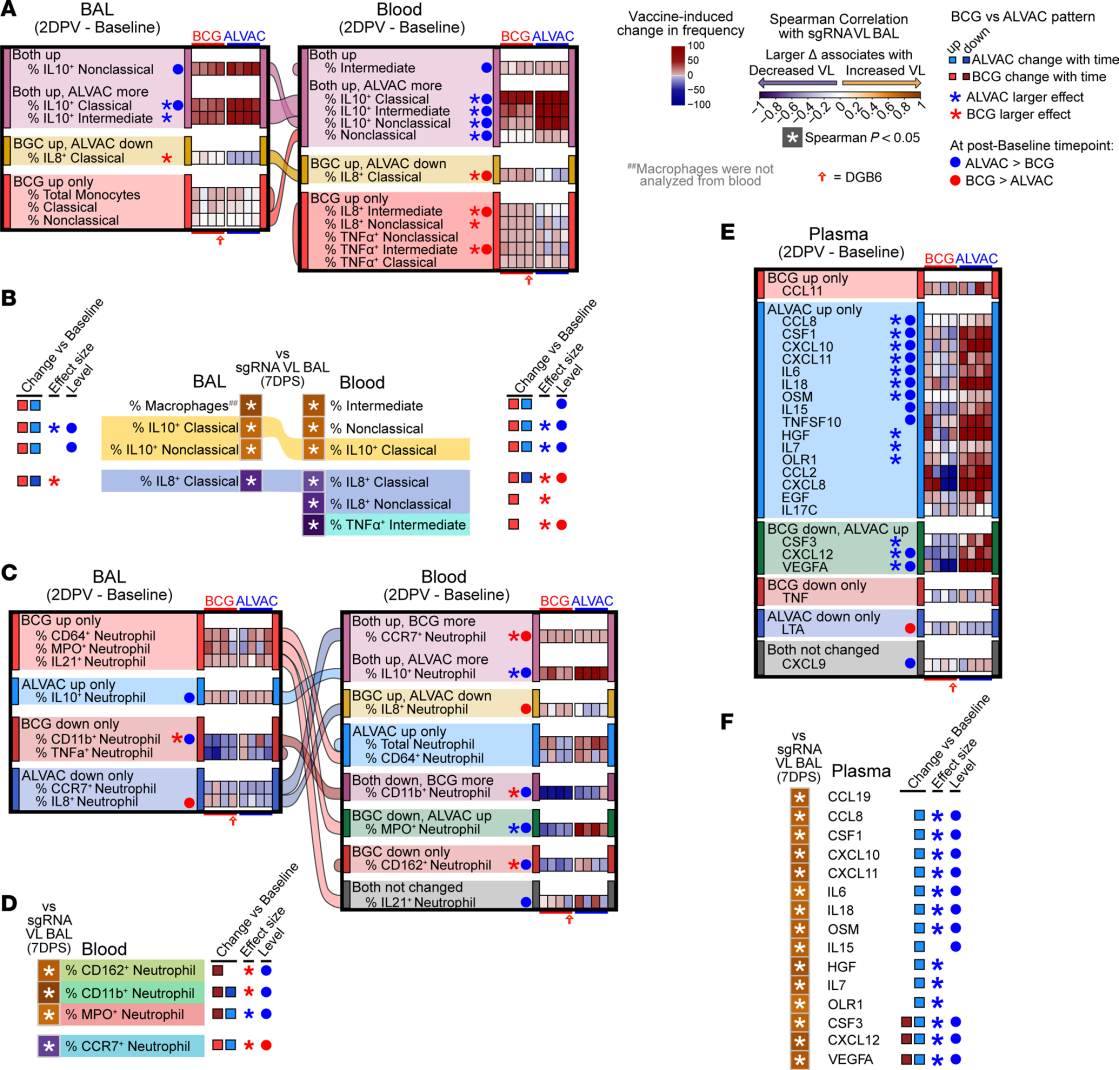
BCG vaccine– and ALVAC/Alum vaccine–induced changes in cell subsets and cytokines. (**A**, **C**, and **E**) Heatmaps depict vaccine-induced changes in monocytes (**A**), neutrophils (**C**), and plasma cytokines/chemokines (**E**), sorted by the change with time patterns at 2DPV relative to baseline for ALVAC/Alum and BCG groups separately; generalized estimating equations, *P* < 0.05. Alluvial flow connects each population across the compartments and is colored according to the pattern in BAL. Populations that had identical patterns in both ALVAC/Alum and BCG animals were omitted from the figure to highlight those populations that differed. Red hollow arrow indicates the BCG-vaccinated animal that did not control virus replication. (**B**, **D**, and **F**) Correlation plots link changes in cell frequencies of monocytes (**B**), neutrophils (**D**), or cytokine/chemokine quantities (**F**) in BAL or blood with replicating VL in BAL at 7 days after SARS-CoV-2 infection; Spearman’s *P* < 0.05, highlighting differences between ALVAC/Alum and BCG. Cell populations or cytokine/chemokines that were not associated (Spearman’s *P* > 0.05) with replicating VL in BAL were omitted from the figure for clarity. Change with time, levels, and magnitude differences between groups are summarized from **A**, **C**, and **E** on the periphery of each plot. Cell frequencies/cytokine quantities (“Level”) or magnitude of changes (“effect size”) that significantly differed between vaccine groups (2-tailed Mann-Whitney *P* < 0.05) are indicated by circles or stars, respectively. Alluvial flow connects each population associated in both compartments and is colored according to the cell/cytokine marker. Eight rhesus macaques were analyzed in this figure: BCG vaccine (*n* = 4), ALVAC/Alum vaccine (*n* = 4).

To investigate the type of BCG-elicited innate immunity associated with the control of SARS-CoV-2 replication in the lung, we first assessed the frequency and functionality of monocytes and neutrophils in blood and BAL (Supplemental Figure 2) at 2 days following the completion of both immunization regimens (2DPV). At the same time point, we also quantified the absolute concentrations of 35 cytokines/chemokines in the plasma. While both vaccination groups increased frequencies of all monocyte subsets producing IL-10 in both blood and BAL by 2DPV relative to their baselines, ALVAC/Alum did so more dramatically than BCG, resulting in higher frequencies of all monocyte subsets producing IL-10 in blood and of classical IL-10^+^ monocytes in BAL (Figure 2A). In contrast, BCG increased while ALVAC/Alum decreased frequencies of classical monocytes producing IL-8 in both compartments, and BCG alone increased blood frequencies of monocyte subsets producing IL-8 or TNF-α (Figure 2A). Strikingly, in blood and BAL, the frequencies of IL-10–producing monocyte subsets were associated with higher levels of replicating SARS-CoV-2 at day 7, whereas monocyte subsets producing IL-8 and TNF-α correlated with lower SARS-CoV-2 replication (Figure 2B and Supplemental Figure 3A). Taken together, these data suggest that BCG induces pro-inflammatory immune responses, which in turn help control viremia in the lungs.

### BCG dampens nonprotective and guides helpful neutrophil subsets in lungs.

Vaccination had complex effects on neutrophil subset populations. As with monocytes, at 2DPV we observed higher frequencies of vaccine-induced IL-10^+^ neutrophils and lower frequencies of vaccine-induced IL-8^+^ neutrophils in both BAL and blood in the ALVAC/Alum group compared with BCG (Figure 2C). In contrast with monocytes, neutrophils producing IL-10 or IL-8 were not associated with SARS-CoV-2 replication. BCG alone depleted BAL CD11b^+^ neutrophils and more dramatically depleted CD11b^+^ neutrophils in the blood (Figure 2C). Blood myeloperoxidase-expressing (MPO^+^) neutrophils increased in the ALVAC/Alum group and decreased in the BCG group, while CD162^+^ neutrophils decreased solely in the BCG group (Figure 2C). Importantly, all 3 of these neutrophil populations, dampened by BCG in blood, were associated with increased SARS-CoV-2 replication (Figure 2D and Supplemental Figure 3B). The population of CCR7^+^ neutrophils decreased in the BAL of the ALVAC/Alum group and increased most dramatically in the blood of the BCG group (Figure 2C), where it was associated with decreased SARS-CoV-2 replication in the lung (Figure 2D and Supplemental Figure 3B).

Interestingly, while neutrophils producing CD64, the high-affinity Fc receptor; MPO, a mediator of pathogen oxygen-dependent killing in the phagosome; and IL-21, a cytokine that orchestrates B cell responses, had elevated frequencies in blood in the ALVAC/Alum group, the frequency of these populations in BAL was enhanced only in the BCG group (Figure 2C). Together, these data suggest that by increasing CCR7, a cell homing receptor to lymph nodes that contributes to initiating adaptive responses, BCG vaccination may have favored homing of CD64^+^, MPO^+^, and CD32^+^ neutrophils to the BAL while diminishing nonprotective CD162^+^, CD11b^+^, and MPO^+^ neutrophil responses in the blood.

ALVAC/Alum vaccination induced a massive cytokine/chemokine response in plasma compared with the BCG group, including CCL8, CSF1, CXCL10, CXCL11, IL-6, IL-18, OSM, IL-15, TNFSF-10, HGF, IL-7, OLR1, CSF3, CXCL12, and VEGFA (Figure 2E). However, with the exception of TNFSF-10, all of these cytokines/chemokines were associated with increased SARS-CoV-2 replication in the lung (Figure 2F and Supplemental Figure 3C), suggesting ALVAC/alum induces a vast array of cytokines, a response not seen with BCG vaccination, which in turn fail to control viremia in the lungs.

### BCG and ALVAC/Alum induce differential immune responses in blood at the time of SARS-CoV-2 exposure.

**Figure 3:**
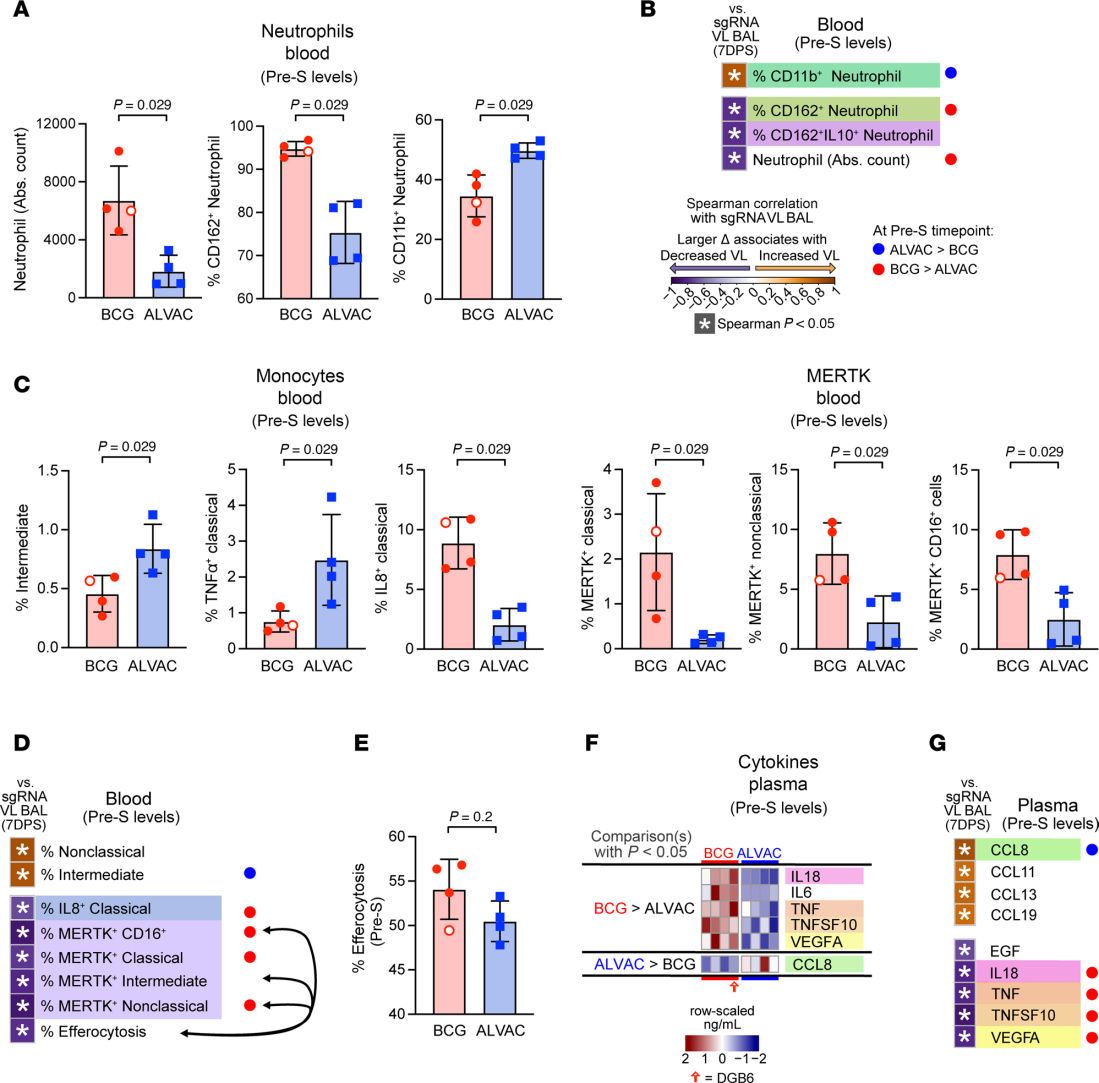
Blood cell subsets and cytokine differences between ALVAC/Alum and BCG pre–SARS-CoV-2 infection. (**A**, **C**, and **E**) Comparison of neutrophil (**A**) or monocyte (**C**) populations, as well as efferocytosis levels (**E**), between ALVAC/Alum and BCG at pre–SARS-CoV-2 (Pre-S). Only cell populations with 2-tailed Mann-Whitney *P* < 0.05 are shown. Bar plots denote mean and error bars are SD. Hollow circle indicates the BCG-vaccinated animal that did not control SARS-CoV-2 replication. (**B**, **D**, and **G**) Correlation analysis shows association of neutrophil (**B**) or monocyte (**D**) population or cytokine/chemokine levels (**G**) at Pre-S with replicating VL in BAL at 7 days after SARS-CoV-2 infection; Spearman’s *P* < 0.05. Cell populations or cytokine/chemokines that were not associated (Spearman’s *P* > 0.05) with replicating VL in BAL were omitted from the figure for clarity. Cell frequencies/cytokine quantities that significantly differed between vaccine groups (2-tailed Mann-Whitney *P* < 0.05) are indicated by circles at right of each plot. Black arrows in **D** indicate trending correlations between efferocytosis and cell population (Spearman’s *P* = 0.069, Supplemental Figure 4C). (**F**) Heatmaps depict row-scaled plasma cytokine/chemokine levels between ALVAC/Alum and BCG, sorted by 2-tailed Mann-Whitney *P* < 0.05 at Pre-S. Only cytokines/chemokines with 2-tailed Mann-Whitney *P* < 0.05 are shown, and colored underlays highlight analytes also in **G**. Eight rhesus macaques were analyzed in this figure: BCG vaccine (*n* = 4), ALVAC/Alum vaccine (*n* = 4).

Next, we evaluated innate immune responses at pre-S in blood only, because no other tissue was collected at this time point to avoid possible lung damage before virus exposure. By pre-S, the absolute number of total neutrophils and CD162^+^ neutrophil frequencies were elevated in the BCG group, whereas CD11b^+^ neutrophil frequencies were elevated in the ALVAC/Alum group (Figure 3A). CD11b^+^ neutrophil frequencies promoted by ALVAC/Alum were associated with increased replicating lung VL, whereas CD162^+^ neutrophils and total neutrophils promoted by BCG associated with decreased replicating VL in the lung (Figure 3B and Supplemental Figure 4A).

At this pre-S time point, intermediate monocytes and TNF-α^+^ classical monocytes were higher in ALVAC/Alum group (Figure 3C) and intermediate monocytes correlated with increased virus replication (Figure 3D and Supplemental Figure 4B), while IL-8^+^ classical monocytes, as well as classical, nonclassical, and total CD16^+^ monocytes expressing MERTK (Figure 3C) were higher in the BCG-vaccinated group and were correlated with decreased virus replication (Figure 3D and Supplemental Figure 4B). Monocyte-mediated efferocytosis is necessary for the clearance of apoptotic cells to maintain tissues’ homeostasis (35). Though efferocytosis appeared comparable between the 2 vaccinated groups (Figure 3E), its frequency was associated with decreased virus replication (Supplemental Figure 4B), and as expected this function was positively associated with nearly all monocyte subsets expressing MERTK (Figure 3E and Supplemental Figure 4C). At pre-S, IL-18, TNF, TNFSF-10, and VEGFA in blood were higher in the BCG group (Figure 3F) and associated with decreased replicating virus in the lung, whereas CCL8, higher in the blood of the ALVAC/Alum group, associated with increased virus replication in lung (Figure 3G and Supplemental Figure 4D).

### BCG increases infiltrating IL-10^+^ neutrophils and IL-10^+^ monocytes, which correlate with lower SARS-CoV-2 replication in lung.

**Figure 4:**
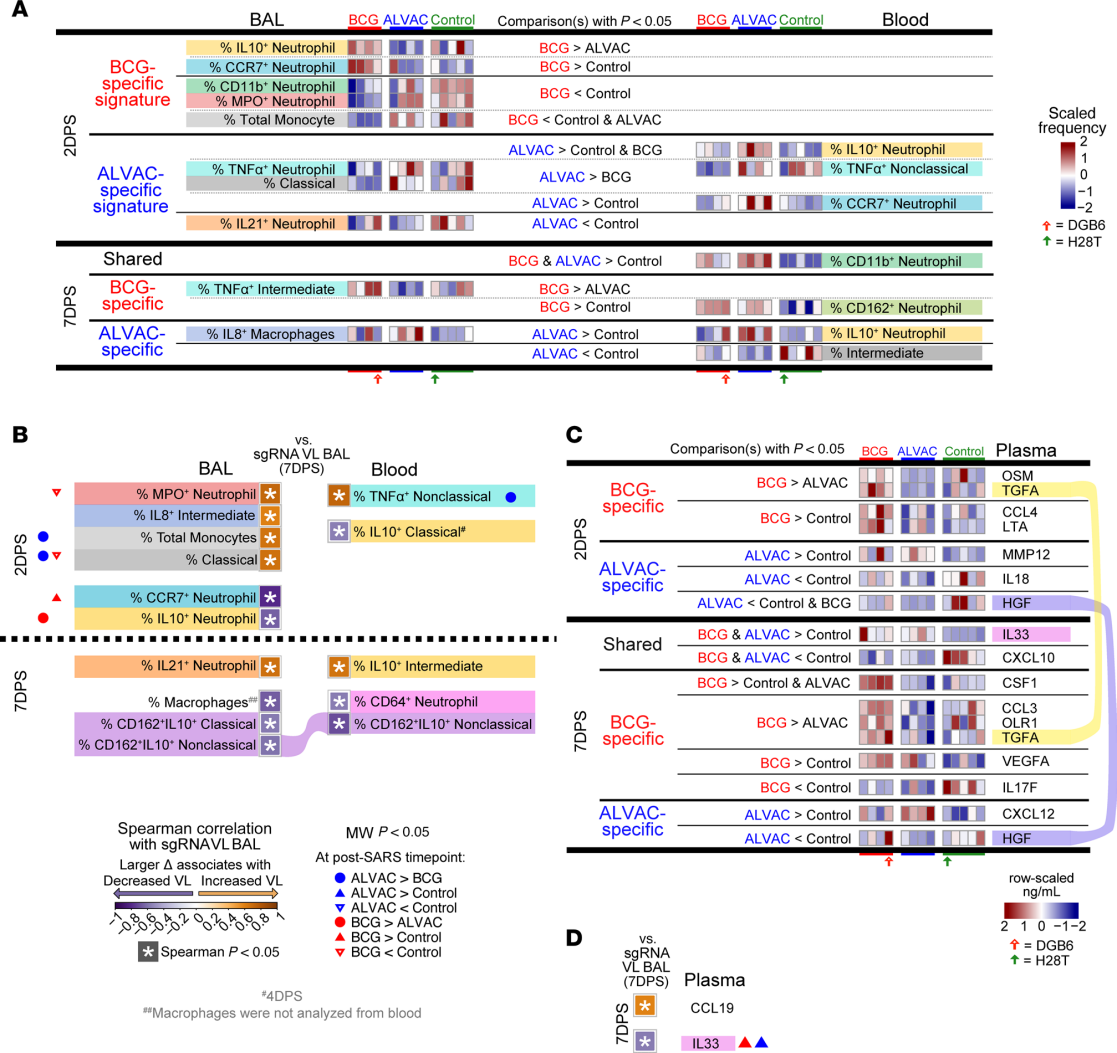
Differences in cell subsets and cytokines between ALVAC/Alum and BCG following SARS-CoV-2 infection. (**A** and **C**) Heatmaps display row-scaled frequencies of cell populations (**A**) in BAL and blood or plasma cytokine levels (**C**) across BCG, ALVAC/Alum, and nonvaccinated control animals, sorted by their significant difference pattern (2-tailed Mann-Whitney *P* < 0.05) at specified postinfection time points. Only cell populations with 2-tailed Mann-Whitney *P* < 0.05 for at least 1 pair of groups are shown. Underlay colors highlight the cell/cytokine marker across time points or compartments. Red and green arrows indicate the BCG-vaccinated animal that did not control virus replication and the nonvaccinated animal that did control virus replication, respectively. (**B** and **D**) Correlation plots link cell frequencies (**B**) and cytokine levels (**D**) at specified postinfection time points with replicating VL in BAL at 7 days after SARS-CoV-2 infection. Differences between groups are summarized from **A** or **C**, indicated by circles (BCG vs. ALVAC/Alum) or triangles (vaccinated vs. nonvaccinated control). Populations that were not significantly different (Mann-Whitney *P* < 0.05) between at least 1 pair of groups were omitted for clarity. Underlay colors highlight the cell/cytokine marker, and alluvial flow connects each population/cytokine associated in both compartments or time points. Thirteen rhesus macaques were analyzed in this figure: BCG vaccine (*n* = 4), ALVAC/Alum vaccine (*n* = 4), and controls (*n* = 5).

Finally, we evaluated the differences between BCG, ALVAC/Alum, and control (nonvaccinated) groups after infection with SARS-CoV-2. Focusing on cell populations in BAL, at 2DPS, IL-10^+^ neutrophils and CCR7^+^ neutrophils were higher in BCG compared with ALVAC/Alum or controls, respectively (Figure 4A), and both of these elevated cell frequencies were associated with lower replicating virus in the lung at 2DPS (Figure 4B and Supplemental Figure 5A). In contrast, BCG depleted MPO^+^ or CD11b^+^ neutrophils at 2DPS in BAL relative to controls and displayed the lowest levels of total monocytes across all 3 groups (Figure 4A), and both CD11b^+^ neutrophils and total monocytes were associated with higher virus replication in BAL (Figure 4B and Supplemental Figure 5A). In blood, there was minimal overlap between populations that differed across treatment groups and those that associated with replicating virus (Figure 4, A and B, and Supplemental Figure 5A). The exception was nonclassical monocytes expressing TNF-α at 2DPS (Figure 4A), which were elevated in ALVAC/Alum compared with BCG and associated with increased replicating SARS-CoV-2 (Figure 4B and Supplemental Figure 5A). Even though IL-10^+^ infiltrating CD162^+^ monocyte subsets did not differ in frequency across the experimental groups after SARS-CoV-2 in either compartment, their frequency in both blood and BAL correlated with decreased virus replication in the lung at 7DPS (Figure 4B and Supplemental Figure 5A).

Similar to the cell populations in blood, the differences in plasma proteomic profile across experimental groups (Figure 4C) largely did not align with the biomarkers associated with viral replication (Figure 4D and Supplemental Figure 5B). In fact, SARS-CoV-2–infected animals at 2DPS and 7DPS displayed only 1 cytokine, IL-33, released by endothelial and epithelial cells following damage caused by pathogens (36), whose levels did not differ between the vaccinated groups but was lower in control animals. Nuclear IL-33 is immediately available to act as an early signifier of damage, through recruitment of neutrophils, eosinophils, and NK cells and by amplifying a type 2 (Th2, ILC2, M2-like macrophage) response in order to initiate wound healing (37, 38). At 7DPS, elevated IL-33 levels correlated with a significantly decreased viral replication (Figure 4D and Supplemental Figure 5B).

### BCG-induced CCR7^+^ Th2 and CCR7^+^ activated memory CD4^+^ cells help control SARS-CoV-2 replication in lung.

**Figure 5:**
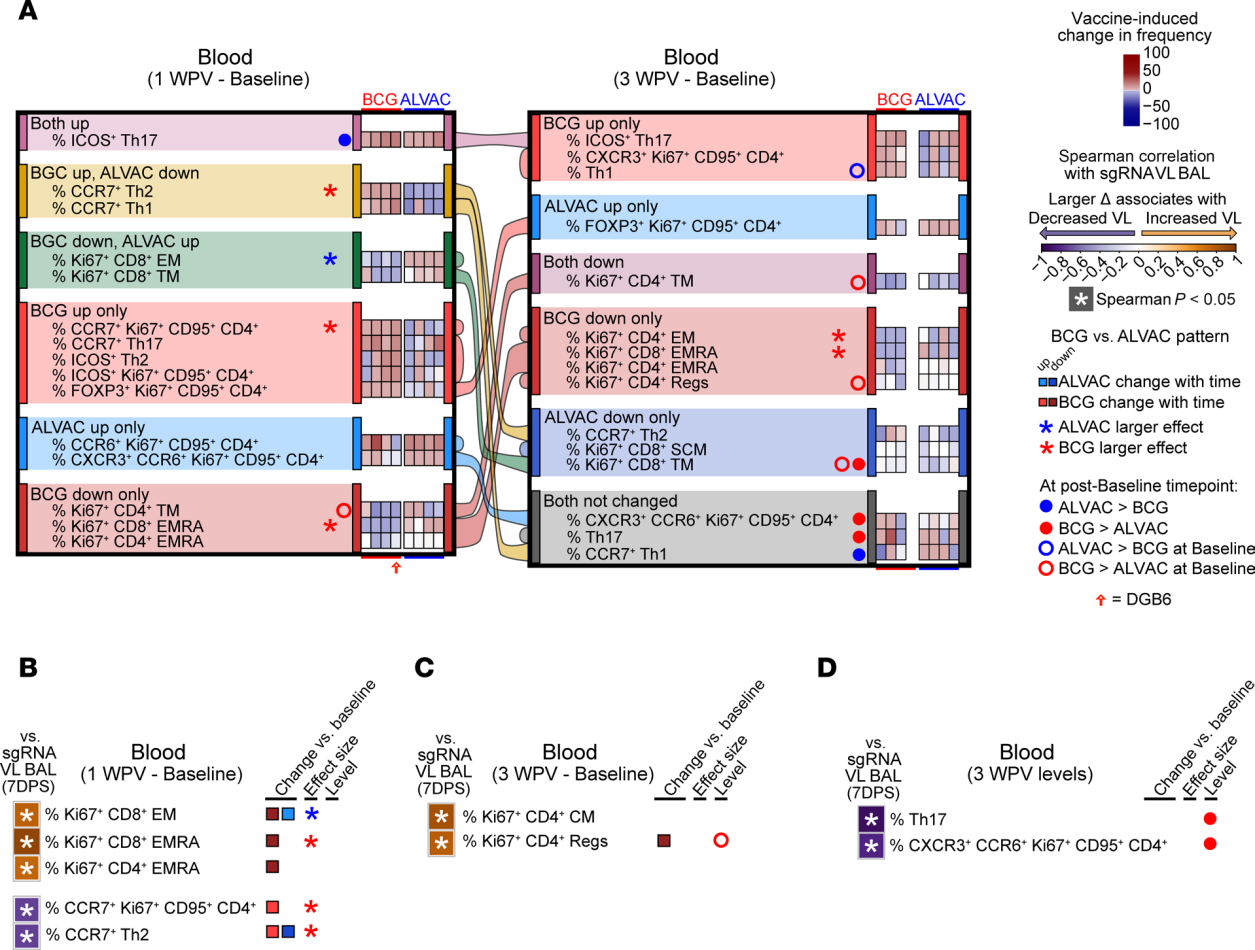
Differences in T cell subsets between ALVAC/Alum and BCG following vaccination. (**A**) Heatmaps depict vaccine-induced changes in T cell populations in blood, sorted by change with time patterns at 1 week (left) or 3 weeks (right) relative to baseline for ALVAC/Alum and BCG groups separately (generalized estimating equations, *P* < 0.05). Alluvial flow connects each population across the time point intervals and is colored according to the pattern at 1 week after vaccination. Populations that had identical patterns in both ALVAC/Alum and BCG animals were omitted from the figure to highlight those populations that differed. Regs, CD4^+^ regulatory T cells; WPV, weeks postvaccination. (**B**–**D**) Correlation plots link vaccine-induced changes in cell frequencies in blood at (**B**) 1 week after vaccination or (**C**) 3 weeks postvaccination or (**D**) levels at 3 weeks postvaccination, with replicating VL in BAL at 7 days after SARS-CoV-2 infection, highlighting differences between ALVAC/Alum and BCG (Spearman’s *P* < 0.05). Cell populations that were not associated (Spearman’s *P* > 0.05) with replicating VL in BAL were omitted from the figure for clarity. Change with time, levels, and magnitude differences between groups are summarized from **A** on the right of each plot. Cell frequencies (“Level”) or magnitude of changes (“effect size”) that significantly differed between vaccine groups (2-tailed Mann-Whitney *P* < 0.05) are indicated by circles or stars, respectively. Open circles indicate differences between the groups at baseline. Eight rhesus macaques were analyzed in this figure: BCG vaccine (*n* = 4 for 1 week postvaccination, *n* = 3 for 3 weeks postvaccination), ALVAC/Alum vaccine (*n* = 4).

Next, we investigated whether the innate responses induced by prior exposure to BCG or ALVAC/Alum affected adaptive CD4^+^ or CD8^+^ T cell response to SARS-CoV-2. We used the Ki67^+^ marker in the gating strategies summarized in Supplemental Figure 6 and quantified blood frequencies of naive T (CD28^+^CCR7^+^CD45RA^+^), central memory (CM; CD28^+^CCR7^+^CD45RA^–^), transitional memory (TM; CD28^+^CCR7^–^CD45RA^–^), effector memory (EM; CD28^-^CCR7^–^CD45RA^–^), RA^+^ effector memory (EMRA; CD28^–^CCR7^–^CD45RA^+^), stem cell memory (SCM; CD95^+^CD28^+^CCR7^+^CD45RA^+^), memory Th1 (Ki67^+^CD95^+^CCR6^–^CXCR3^+^CD4^+^), memory Th2 (Ki67^+^CD95^+^CCR6^–^CXCR3^–^CD4^+^), and memory Th17 (Ki67^+^CD95^+^CCR6^+^CXCR3^–^CD4^+^) cells. At 1 week postvaccination, the BCG group increased CCR7^+^ Th1 and Th2 cells while they were decreased by ALVAC/Alum (Figure 5A). Increases in CCR7^+^ Th2 frequency correlated with reduced viral load in the lung (Figure 5B and Supplemental Figure 5C, top). BCG vaccination also enhanced CCR7-producing Ki67^+^CD95^+^CD4^+^ cells (Figure 5A), and these elevated frequencies were associated with decreased viremia in the lung (Figure 5B and Supplemental Figure 5C, top).

In contrast, ALVAC/Alum increased and BCG decreased frequencies of Ki67^+^CD8^+^ EM, while only BCG depleted CD4^+^ and CD8^+^ EMRA (Figure 5A). All of these populations, dampened by BCG, were associated with increased virus in the lungs (Figure 5B and Supplemental Figure 5C, top). While the BCG group had higher baseline levels of Ki67^+^CD4^+^ Regs than the ALVAC/Alum group, they were reduced in the BCG alone group by week 3 postvaccination (Figure 5A), and their decrease in frequency was associated with SARS-CoV-2 control (Figure 5C and Supplemental Figure 5C, middle). Finally, at 3 weeks postvaccination, BCG displayed higher frequencies of Th17 and CXCR3^+^CCR6^+^Ki67^+^CD95^+^CD4^+^ cells compared with the ALVAC/Alum group. These population frequencies were also strongly correlated with decreased virus level in the lung at 7DPS (Figure 5D and Supplemental Figure 5C, bottom). This finding suggests an important role of T cells in protecting against SARS-CoV-2, which have previously been observed to be elevated in humans exposed to BCG (39, 40).

### SARS-CoV-2 infection generates binding antibody by 7 days of exposure.

Analyses of binding IgG and IgM antibody titers against spike and receptor binding domain protein of SARS-CoV-2 ancestral wild-type variants showed comparable responses among the different groups of animals at 14DPS (Supplemental Figure 7), demonstrating that prior exposure to BCG or ALVAC/Alum did not hasten anti–SARS-CoV-2 B cell responses. Similarly, 2 weeks following infection, neutralizing antibodies were undetectable in all groups (Supplemental Table 1). These data suggest that the lower level of virus replication in the lung of BCG-vaccinated animals was due to innate immune responses.

## Discussion

Growing evidence points to the importance of innate immune responses as a critical line of defense against infectious disease (41). Attenuated BCG has been used to immunize a vast population around the globe, and it has been reported to induce innate response that protects against diverse infections in different epidemiological, preclinical, and clinical studies (4–8, 19, 21, 22). Although prior exposure to BCG has been reported to reduce influenza A virus–related morbidity and mortality, its role in protecting against SARS-CoV-2 morbidity remains debated (42, 43). BCG vaccination failed to protect against SARS-CoV-2 infection in a case control study in Québec, Canada (44), as well as in care providers in South Africa (45). Interestingly, the mRNA vaccines that are widely used against SARS-CoV-2 (2, 3) also do not protect most people against infection, but they reduce COVID-19 severity. Fu et al. used data from the Johns Hopkins University Coronavirus Resource Center, BCG program data from the World Atlas of BCG Policies and Practices, and WHO/UNICEF data to establish a dynamic model that showed that BCG vaccination plays a protective role against COVID-19 (46). For instance, it has been shown that intravenous BCG vaccination protects human ACE2-transgenic mice against lethal dose of SARS-CoV-2 infection by modulating lung innate immune responses (47). Moreover, combination of SARS-CoV-2 spike antigen with BCG accelerates the production of virus-specific IgG antibodies in vaccinated mice, contributing to protection against disease following viral challenge (48). Taken together, these studies suggested that BCG vaccination might play a protective role in reducing the severity of SARS-CoV-2 infection.

**Figure 6:**
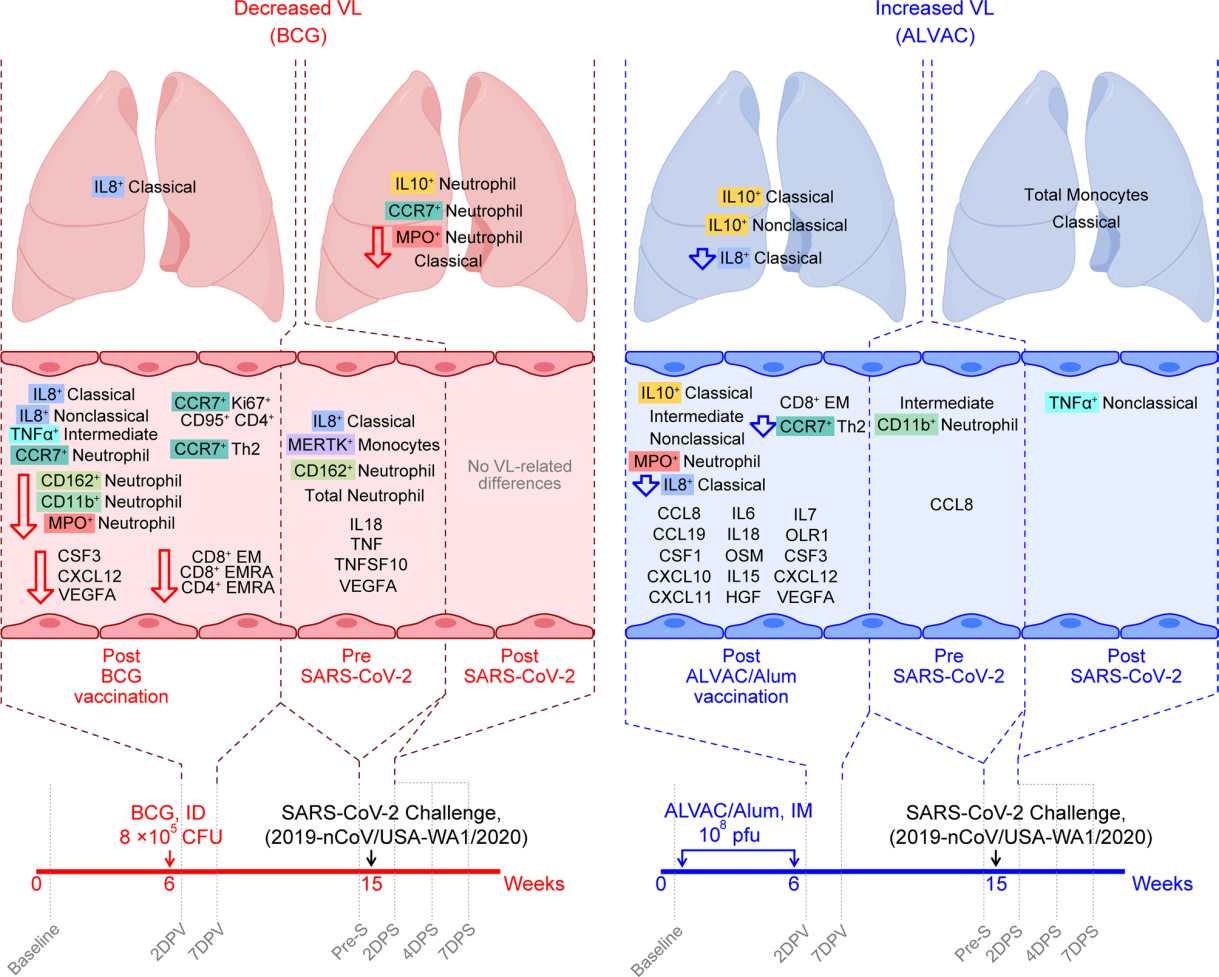
Summary of differences in immune responses induced by BCG and ALVAC/Alum and their association with replicating SARS-CoV-2 in BAL. BCG-specific (left, pink) and ALVAC-specific (right, blue) immune responses are associated with reduced or increased VL, respectively. Cell populations and cytokines associated with replicating VL and differing between BCG and ALVAC/Alum are summarized by time (left to right) in the compartments indicated. Hollow down arrows specify cell populations/cytokines that are selectively reduced in the vaccine group indicated.

As BCG (7, 8, 49–53) and ALVAC/Alum-based vaccines (9, 28, 54) have been shown to induce differential pro- and antiinflammatory based trained immunity, respectively, we analyzed the innate immune responses elicited by BCG and contrasted them with the innate immunity elicited by ALVAC/Alum immunization in providing protection against SARS-CoV-2 replication in the lung, as a surrogate for COVID-19 disease. In this study, we observed that BCG vaccination increased IL-8^+^ classical monocytes in blood and BAL, as well as IL-8^+^ nonclassical monocytes and MERTK^+^ monocytes in blood, which are associated with efferocytosis, correlated with decreased viral replication (Figure 6 and Supplemental Figure 8). Moreover, BCG-induced blood neutrophils and Th2 and Ki67^+^CD95^+^CD4^+^ T cells, all producing CCR7, were also associated with decreased replicating VL. Furthermore, BCG vaccination reduced blood frequencies of neutrophils producing CD162, CD11b, and MPO; CD8^+^ EM cells; and CD8^+^ and CD4^+^ EMRA cells, as well as plasma levels of CSF3, CXCL12, and VEGFA, and all of these depletions were linked to viral control. In contrast, ALVAC/Alum vaccination elevated IL-10^+^ classical monocytes in the blood and BAL, IL-10^+^ nonclassical monocytes in BAL, MPO^+^ neutrophils in blood, and levels of CSF3, CXCL12, VEGFA, CXCL11, and many other cytokines in plasma, all associated with enhanced viral replication. Additionally, ALVAC/Alum depleted IL-8^+^ classical monocytes in both blood and BAL, as well as CCR7^+^ CD95^+^ Th2 cells in blood, and these depletions correlated with increased viral replication (Figure 6 and Supplemental Figure 9). Although BCG vaccination did not prevent virus acquisition, it effectively engaged neutrophils and monocytes mediating efferocytosis, which was crucial for containing early viral replication in the lung. Taken together, these findings suggest that vaccines generating pro-inflammatory trained immunity might induce an immune profile favorable to SARS-CoV-2 viral containment, supporting data observed in humans (55).

We characterized immune cell populations obtained from blood and BAL, the latter of which predominantly reflects the epithelial surface of the lung. Both BCG and ALVAC/Alum vaccines were found to engage neutrophils, monocytes, and macrophages. Notably, functional assays such as efferocytosis, which was performed using blood-derived cells, could not be performed on BAL samples because of the limited cellular yield from these samples. Since the low number of animals in each vaccine group constituted a limitation of this study, data from both the BCG-vaccinated (*n* = 4) and ALVAC/Alum-vaccinated (*n* = 4) animals were combined to perform correlation analyses. Although correlative analyses of innate immune responses in the lung and blood suggest a potential protective role, the mechanisms by which innate immunity limits SARS-CoV-2 replication needs further study. Targeted depletion studies in mouse models — such as those focusing on IL-8^+^ monocytes, IL-10^+^ neutrophils, or CCR7^+^ neutrophils — might offer valuable mechanistic insights into the contribution of innate immune cells to the protective effects associated with BCG exposure.

The vaccines used in this study were not designed to generate binding antibodies against SARS-CoV-2 or antigen-specific T cell responses, as the vaccines were not SARS-CoV-2 antigen specific. Aerosol administration of BCG in rhesus macaques, followed by SARS-CoV-2 challenge, resulted in the induction of CD14^+^ classical monocytes and Vδ2 γδ T cells, suggesting a BCG-mediated priming effect on innate immune and nonconventional T cell populations (53). Thus, we tested whether the T cell and antibody responses elicited by the vaccination could provide any crossreactivity with SARS-CoV-2 and confer protection from infection. Interestingly, we observed that Th2 cells and Ki67^+^CD95^+^CD4^+^ cells producing CCR7 were associated with decreased viral load, suggesting potential crossreactive T cell responses. Additionally, the SARS-CoV-2–specific binding antibodies observed in this study were induced by SARS-CoV-2 infection. The observation that BCG vaccination does not enhance SARS-CoV-2 crossreactive humoral immune responses (53) may explain the lack of differences in antibody binding among the animal groups. In individuals infected with SARS-CoV-2, neutralizing antibody (NAb) levels remain low during the first 7 to 10 days after symptom onset, followed by a gradual increase over the subsequent 2 to 3 weeks. The median time to reach a peak of NAb levels is approximately 33 days after symptom onset, with a reported range of 24 to 59 days (56). Similarly, macaques experimentally infected with SARS-CoV-2 exhibited low neutralization titers, with most animals showing titers below 100 against pseudovirus by 35 days postinfection (57). In our current study, we did not detect NAb up to day 14 postinfection; however, binding antibodies were observed by day 14. This suggests that NAb responses may require more time to develop in these animals.

By profiling innate immune cell populations in peripheral blood and BAL, we investigated the effect of immune responses elicited by BCG and ALVAC/Alum vaccination on SARS-CoV-2 infection. This analysis enabled the characterization of temporal dynamics among innate immune cell subsets and their potential roles in the progression of COVID-19 in vaccine-induced pro- and antiinflammatory immune environments. Our findings are consistent with epidemiological and clinical observations from BCG-vaccinated human cohorts (4–6, 25), providing a potential mechanism for how BCG-induced pro-inflammatory innate immunity contributes to the control of SARS-CoV-2 replication.

## Methods

### Sex as a biological variable.

This study was exploratory in nature. To reduce biological variability and because of the limited number of subjects, only male macaques were included. Despite the small sample size, the observed group differences were pronounced, permitting the extraction of meaningful conclusions. Future studies incorporating female macaques will be necessary to determine whether these findings could be generalizable across sexes.

### Animals.

Thirteen male Indian rhesus macaques obtained from the free-range breeding colony on Morgan Island, South Carolina, USA, were used in this study. Ten naive macaques, aged 3 to 4 years at study initiation, were negative for SIV, simian retrovirus, simian T cell leukemia virus (STLV), and SARS-CoV-2. The 10 animals were divided into 3 groups; 4 animals received ALVAC/Alum vaccine, 4 animals received BCG vaccine, and 2 animals remained naive as control group.

Three additional macaques were included as a control group that were previously vaccinated with an anti-SIV vaccine and exposed to SIV challenges. However, they have maintained a negative status for SIV, simian retrovirus, STLV, and SARS-CoV-2 for over 8 months following the last treatment. Of these 3 control animals, 2 were vaccinated at weeks 0 and 4 with DNA encoding SIVgp160 (2 mg/dose) and SIV239gag (1 mg/dose). At 8 weeks the macaques were administered ALVAC encoding *gag/pro/env* (wild-type *env*), 10^8^ pfu/dose in 1 mL PBS. At week 12 the macaques were boosted with the same ALVAC plus SIVgp120 protein (400 μg/dose in 500 µL PBS plus 500 µL 2% Alhydrogel) (InvivoGen, San Diego, California, USA). They were challenged 33 times with SIVmac251.

The other animal was vaccinated at weeks 0 and 4 with DNA encoding SIVgp160ΔV1 (2 mg/dose) and SIV239gag (1 mg/dose). At 8 weeks the macaques were administered ALVAC encoding *gag/pro/env* (wild-type *env*), 10^8^ pfu/dose in 1 mL PBS. At week 12 the macaques were boosted with the same ALVAC plus SIVgp120ΔV1 protein (400 μg/dose in 500 μL PBS plus 500 μL of 2% Alhydrogel (InvivoGen). The animal was challenged 11 times with SIVmac251.

### Immunization and challenge.

Macaques in the ALVAC/alum group were immunized IM at weeks 0 and 6 with ALVAC vector in the right thigh, 10^8^ pfu/dose in 1 mL PBS, and in the left thigh the animals received 1 mL of 2% Alum hydrogel. Macaques in the BCG group were immunized 1 time with BCG vaccine 8 ×10^6^ CFU ID in the thigh.

Eight weeks postvaccination, the animals were transferred to the Bioqual facility located in Rockville, Maryland, USA, where they were all individually housed. All the animals were challenged with 2 × 10^6^ pfu 2019-nCoV/USA-WA1/2020 SARS-CoV-2 virus (BEI NR-52281) by the intranasal and intratracheal routes in a total volume of 2 mL. Following challenge, viral loads were assessed in BAL and oropharyngeal swab and nasal swab samples by real-time PCR (RT-PCR) for total VL and sgRNA. Viral RNA was quantified using an RT-PCR assay targeting the SARS-CoV-2 envelope and nucleocapsid genes. Animals were sacrificed 14 days following viral challenge. Immunologic and virologic assays were performed blinded. All animal studies were conducted in compliance with all relevant local, state, and federal regulations and were approved by the Bioqual Institutional Animal Care and Use Committee.

### RNA extraction and quantitative PCR quantification.

Viral RNA was isolated from nasal swab, oropharyngeal swabs, and the BAL collected from macaques either prechallenge or 2, 4, or 7 days postchallenge using the QIAamp Viral RNA Mini Kit (QIAGEN, Valencia, California, USA) following manufacturer’s instructions. Briefly, the swabs were immersed in PBS, vigorously vortexed, and incubated for 10 minutes at room temperature (RT). Swabs were then wrung out and discarded. Then 140 μL of the swab’s remaining solution or BAL was incubated with 560 μL of the lysis buffer in the presence of carrier RNA for 10 minutes at RT and then mixed with 560 μL of ethanol. The mix was then transferred into the spin columns and centrifuged at 6,000*g* for 1 minute. Next, the columns were washed twice with 500 μL of ethanol-based buffers. The RNA was then eluted in either 20 μL (swabs) or 50 μL (BAL) of Ultrapure water and stored at –80°C until used.

The TaqPath 1-Step RT-qPCR Master Mix, CG, was used for reverse transcription and RT-PCR (Thermo Fisher Scientific, Waltham, Massachusetts, USA; catalog A15300). VLs were analyzed by measuring the expression of both the nucleocapsid and the envelope genes. The following primers were used: nucleocapsid gene (forward: 5′-CGATCTCTTGTAGATCTGTTCTC-3′, reverse 5′-GGTGAACCAAGACGCAGTAT-3′; probe: 5′-6FAM-TAACCAGAATGGAGAACGCAGTGGG-BHQ1a-3′); envelope gene (forward: 5′-CGATCTCTTGTAGATCTGTTCTC-3′, reverse 5′-ATATTGCAGCAGTACGCACACA-3′; probe: 5′-AminoC6-Rox-ACACTAGCCATCCTTACTGCGCTTCG-BHQ2a-3′). Each reaction contained 5 μL of Master Mix, 10 μM of forward and reverse primers, 5 μM of labeled probe, and 100 ng of RNA in a volume of 20 μL/reaction. Samples were run in duplicates on a Rotor-Gene Q (QIAGEN) instrument using the following program: i) 25°C for 2 minutes, ii) 50°C for 15 minutes, iii) 95°C for 2 minutes, and iv) 40 cycles of 95°C for 3 seconds and 30 seconds at 60°C. A 10-fold serial dilution of a plasmid was used to generate standard curves. The series contained 10^8^ to 10 copies/reaction with each level run in duplicate. A standard curve was included in every run. Nucleocapsid and envelope gene viral load/reaction was calculated by interpolation to the respective standard curve.

### Analysis of neutrophils, monocytes, and macrophages in blood and BAL.

The frequency and the cytokine levels of neutrophils, monocytes, and macrophages were measured in the blood and BAL at prevaccination, 48 hours after last vaccination, pre-S, and 2, 4, and 7 days after SARS-CoV-2 exposure. Freshly collected 200 μL of whole blood was used for the experiment. Additionally, BAL samples were centrifuged at 1,300*g* for 7 minutes, and a portion of the pelleted cells was used for the experiment. Cells were stained with Live/Dead blue dye (catalog L34961, 0.5 μL) from Thermo Fisher Scientific, followed by surface staining with the following: Alexa Fluor 700 anti-CD3 (SP34-2; catalog 557917, 5 μL), Alexa Fluor 700 anti-CD20 (2H7; catalog 560631, 5 μL), Alexa Fluor 700 anti-CD8 (RPA-T8; catalog 565165, 5 μL), BV510 anti-CD11c (3.9; catalog 748269, 5 μL), BB700 anti-CD162 (KPL-1; catalog 745768, 5 μL), BV786 anti-CD45 (D058-1283; catalog 563861, 5 μL), BUV395 anti-CD123 (7G3; catalog 564195, 5 μL), BUV496 anti-CD16 (3G8; catalog 612944, 5 μL), BUV563 anti-CD32 (FLI8.26; catalog 741368, 5 μL), BUV661 anti–HLA-DR (G46-6; catalog 612980, 5 μL), BUV737 anti-CD11b (ICRF44; catalog 741826, 5 μL), BUV805 anti-CD14 (M5E2; catalog 565779, 5 μL), PE-CF594 anti-CD64 (10.1; catalog 565389, 5 μL) from BD Biosciences (San Jose, California, USA); FITC anti-CD66abce (TET2; catalog 130-116-522, 5 μL) from Miltenyi Biotec (Gaithersburg, Maryland, USA); and APC-eFluor780 anti-CCR7 (3d12; catalog 47-1979-42, 5 μL) from Thermo Fisher Scientific for 30 minutes at room temperature. This was followed by permeabilization with a FOX3 transcription buffer set (catalog 00-5523-00) from eBioscience (San Diego, California, USA) according to the manufacturer’s recommendation and subsequently intracellular staining with the following: PE anti-MPO (MPO455-8E6; catalog 12-1299-42, 5 μL) from Thermo Fisher Scientific and Alexa Fluor 647 anti- IL-21 (3A3-N2.1, catalog 560493, 5 μL), PE-Cy7 anti–TNF-α (MAb11, catalog 557647, 5 μL), BV421 anti–IL-8 (G265-8, catalog 563310, 5 μL), and BV650 anti–IL-10 (JES3-9D7, catalog 564051, 5 μL) from BD Biosciences for 30 minutes at RT. Samples were acquired on a BD Biosciences FACSymphony A5 cytometer and analyzed with FlowJo software 10.6 (TreeStar, Ashland, Oregon, USA). Neutrophils were gated as singlets, live cells, CD45^+^ cells, or CD3^–^, CD20^–^, CD8^–^, CD123^–^, CD11c^–^, CD14^–^, CD16^–^, and CD66abce^+^ cells. Macrophages were gated as singlets, live cells, CD45^+^ cells, or CD3^–^, CD20^–^, CD11b^+^, HLA-DR^+^, FSC-A^hi^, and SSC-A^hi^ cells. Monocytes were gated as singlets, live cells, CD45^+^ cells, or CD3^–^, CD20^–^, CD11b^+^, HLA-DR^+^, FSC-A^lo^, SSC-A^lo^, CD14^+/–^, and CD16^+/–^ cells. Cytokines were gated on the parent population.

### MERTK^+^ monocytes in blood.

The frequency of MERTK^+^ monocytes was measured in macaque cryopreserved PBMCs at prevaccination and 6 weeks after last vaccination. PBMCs were stained with Live/Dead blue dye (0.5 μL) from Thermo Fisher Scientific, followed by surface staining with the following: Alexa Fluor 700 anti-CD3 (SP34-2; catalog 557917, 5 μL), Alexa 700 Fluor anti-CD20 (2H7; catalog 560631, 5 μL), BUV496 anti-CD16 (3G8; catalog 612944, 5 μL), BUV661 anti–HLA-DR (G46-6; catalog 612980, 5 μL), BUV737 anti-CD11b (ICRF44; catalog 741826, 5 μL), BUV805 anti-CD14 (M5E2; catalog 565779, 5 μL), and BV786 anti-CD45 (D058-1283; catalog 563861, 5 μL) from BD Biosciences and PE anti-MERTK (590H11G1E3; catalog 367608, 5 μL) from BioLegend (San Diego, California, USA) for 30 minutes at RT. Samples were acquired on a BD Biosciences FACSymphony A5 cytometer and analyzed with FlowJo software 10.6. Monocytes were gated as singlets, live cells, CD45^+^ cells, and CD3^–^, CD20^–^, CD11b^+^, HLA-DR^+^, FSC-A^lo^, SSC-A^lo^, CD14^+/–^, and CD16^+/–^ cells.

### Efferocytosis assay.

The frequency of efferocytotic CD14^+^ cells was assessed by Efferocytosis Assay Kit (catalog 601770, Cayman Chemical Company, Ann Arbor, Michigan, USA). CD14^+^ cells were used as effector cells, whereas apoptotic neutrophils were used as target cells. The protocol was readapted in order to use CD14^+^ monocyte cells rather than differentiated macrophages due to the low cell availability. CD14^+^ cells were isolated from cryopreserved PBMCs (10 × 10^6^ cells) collected following prestudy and 8 weeks after last immunization (week 14) by using nonhuman primate CD14 MicroBeads (130-091-097, Miltenyi Biotec) following manufacturer instructions. At the end of the separation, cells were counted and stained with CytoTell Blue (AAT Bioquest, Sunnyvale, California, USA) provided by the kit and following manufacturer instructions. One unrelated macaque was used as source of neutrophils as target cells. Neutrophils were isolated as previously described. Briefly, following isolation of PBMCs by Ficoll-Paque (GE Healthcare, now Cytiva, Chicago, Illinois, USA), the cellular pellet was added to an equal volume of 20% dextran in water, gently mixed, and incubated for 1 minute. Approximately 3 volumes of PBS were added, mixed again, and incubated in the dark for 50–60 minutes. At the end of incubation, the clear layer at the top of the tube containing neutrophils was collected. Cells were pelleted and treated with ACK lysing buffer (Quality Biological, Gaithersburg, Maryland, USA) for 5 minutes at 37°C, washed with R10, and counted. Neutrophils were stained with CFSE provided by the kit following manufacturer instructions. The apoptosis of neutrophils was induced by treatment with Staurosporine Apoptosis inducer provided by the kit. Briefly, isolated cells were resuspended in R10 containing Staurosporine diluted 1:1,000 and incubated at 37°C for 3 hours. At the end of the incubation, cells were washed twice with R10 and used for the efferocytosis assay. Subsequently, effector and apoptotic target cells were cultured alone (as controls) or cocultured at a ratio of 1 effector CD14^+^ cell to 3 target apoptotic neutrophils. Cells were incubated at 37°C for 12 hours. At the end of the coculture, cells were washed with PBS, fixed with 1% paraformaldehyde (PFA) in PBS, and acquired on a FACSymphony A5 and examined using FACSDiva software (BD Biosciences) by acquiring all stained cells. Data were further analyzed using FlowJo 10.6. The frequency of efferocytotic CD14^+^ cells was determined as the frequency of double-positive cells for CytoTell Blue and CFSE on the CytoTell Blue–positive monocytes.

### CD4^+^ and CD8^+^ T cell phenotypes.

The levels of CD4^+^ and CD8^+^ T cell subsets were measured in blood at baseline and at 1, 3, and 4 weeks following last immunization. Staining was conducted on 10^6^ PBMCs. Cryopreserved PBMCs were thawed in R10 and washed once with PBS 1×. Cells were first incubated for 15 minutes at RT with LIVE/DEAD Fixable Blue Dead Cell Stain (1 µL, catalog L23105, Thermo Fisher Scientific). Following incubation, cells were added with surface antibodies BV421 anti-CXCR3 (1C6; catalog 562558, 5 μL) from BD Biosciences, PE-Cy7 anti-CCR6 (G034E3; catalog 353418, 5 μL) from BioLegend, and PerCP-eFluor710 anti-CCR7 (3D12; catalog 46-1979-42, 5 μL) from eBioscience and incubated for 20 minutes at 37°C. Following incubation, cells were then added with the following surface antibodies: PE-CF594 anti-CD25 (BC96, catalog 567489, 5 μL), PE-Cy5 anti-CD95 (DX2; catalog 559773, 8 μL), Alexa Fluor 700 anti-CD3 (SP34-2; catalog 557917, 5 μL), BV750 anti-CD4 (L200; catalog 747202, 5 μL), BV786 anti-CD45 (D058-1283; catalog 563861, 5 μL), BUV395 anti-ICOS (C398.4A; catalog 565884, 5 μL), BUV496 anti-CD8 (RPA-T8; catalog 612942, 5 μL), BUV563 anti-CD45RA (5H9; catalog 741411, 5 μL), and BUV737 anti-CD28 (CD28.2; catalog 612815, 5 μL) from BD Biosciences and APC anti-CD127 (A019D5; catalog 351316, 5 μL) from BioLegend. They were incubated for 30 minutes at RT. Following incubation, cells were washed once with PBS 1× and permeabilized with the FOXP3 transcription buffer set (catalog 00-5523-00) from eBioscience and following manufacturer’s instructions. Cells were subsequently intracellularly stained with antibodies PE anti-FoxP3 (236A/E7; catalog 560852, 5 μL) and FITC anti-Ki67 (B56; catalog 556026, 5 μL) from BD Biosciences and incubated 45 minutes at RT. Samples were washed once, resuspended in 1% PFA, acquired on a BD FACSymphony A5 cytometer, and analyzed with FlowJo software 10.6. Gating was done on live CD45^+^, CD3^+^, CD4^+^CD8^–^, and CD4^–^CD8^+^ T cells. Vaccine-induced CD4^+^ cells were identified as CD95^+^Ki67^+^, whereas CXCR3 and CCR6 expression was used to identify Th1 (CCR6^–^CXCR3^+^), Th2 (CCR6^–^CXCR3^–^), or Th17 (CCR6^+^CXCR3^–^) CD4^+^ populations. Additionally, subsets of CD8^+^ and CD4^+^ T cells were analyzed based on the expression of CD95, CD28, CCR7, and CD45RA. In particular cells were defined as naive (CD28^+^CCR7^+^CD45RA^+^), CM (CD28^+^CCR7^+^CD45RA^–^), TM (CD28^+^CCR7^–^CD45RA^–^), EM (CD28^–^CCR7^–^CD45RA^–^), and EMRA (CD28^–^CCR7^–^CD45RA^+^) (58) or as naive (CD95^–^CD28^+^CCR7^+^CD45RA^+^), SCM (CD95^+^CD28^+^CCR7^+^CD45RA^+^), CM (CD95^+^CD28^+^CCR7^+^CD45RA^–^), TM (CD95^+^CD28^+^CCR7^–^CD45RA^+^), and EM (CD95^+^CD28^–^CCR7^–^CD45RA^–^) (40).

### Plaque reduction neutralization tests.

To measure neutralization, serum from each animal was diluted to 1:10 followed by a 3-fold serial dilution. Diluted samples were then incubated with 30 pfu of wild-type 2019-nCoV/USA-WA1/2020 SARS-CoV-2 (BEI NR-52281) in an equal volume of culture medium for 1 hour at 37°C. The serum-virus mixtures were added to a monolayer of confluent Vero E6 cells (ATCC, catalog CRL-1586) in duplicate wells and incubated for 1 hour at 37°C in 5% CO_2_. Each well was next overlaid with culture medium containing 0.5% methylcellulose and incubated for 3 days at 37°C in 5% CO_2_. The plates were then fixed with methanol at –20°C for 30 minutes and stained with 0.2% crystal violet for 30 minutes at RT. The plates were finally washed once with distilled H_2_O and were left to dry for at least 15 minutes. The plaques in each well were recorded, and the IC_50_ titers were calculated based on the average number of plaques detected in the virus control wells.

### 4-plex Meso Scale Discovery SARS-CoV-2 serology assay.

COVID-19 serology testing was performed on Meso Scale Discovery (MSD; Rockville, Maryland, USA) 384-well 4-plex plates (catalog K25392U) as previously described (59). Briefly, precoated plates with SARS-CoV-2 ancestral wild-type proteins (spike S-2P, receptor binding domain, and nucleoprotein) and BSA were blocked with MSD blocker A solution for 60 minutes at RT, followed by washing. Heat-inactivated samples (60 minutes at 56°C) were added to the plates in duplicate with reference standard and positive controls in an 8-point dilution series. Plates were incubated at RT for 4 hours on a plate shaker at 1,350 rpm. Plates were then washed to remove any unbound antibodies, followed by addition of MSD SULFO-TAG anti-IgG detection antibody. Plates were incubated for 60 minutes at RT on a plate shaker. Plates were washed, and MSD GOLD read buffer with electrochemiluminescence substrate was added to the wells. The plates were read on the MSD MESO Sector S 600 detection system. Data were analyzed on MSD Discovery Workbench software. Samples were assigned arbitrary units (AU/mL) by interpolating to the MSD-provided reference standard. Lower limits of quantitation (LLOQs) were set as 199 AU/mL (spike), 1870 AU/mL (nucleoprotein), and 212 AU/mL (receptor binding domain).

### Proximity Extension Assay on plasma samples.

Protein quantification was executed employing the Olink Target 48 Cytokine panel (Olink Proteomics AB, Uppsala, Sweden) in accordance with the manufacturer’s protocols. This method leverages the Proximity Extension Assay (PEA) technology, as extensively detailed by Assarsson et al. (60). This specific PEA methodology enables the concurrent assessment of 45 distinct analytes. Briefly, we used pairs of oligonucleotide-labeled antibody probes, each tailored to selectively bind to their designated protein targets. Probe/pairs were incubated with 1 µL of plasma. Probes that encountered their cognate proteins are then in close spatial proximity, and their respective oligonucleotides engage in pairwise hybridization. A DNA polymerase was used to amplify the polymerized DNA and to create distinct PCR target sequences. Subsequently we detected and quantified these newly formed DNA sequences through utilization of a microfluidic RT-PCR platform, specifically the Biomark HD system by Fluidigm (Olink Proteomics AB Signature Q100 instrument). Data validation to uphold data integrity was conducted with the Proteomics AB NPX Signature software specifically designed for the Olink analysis: the application was used to import data from the Olink Signature Q100 instrument and process the data. Data normalization procedures were executed employing an internal extension control and calibrators, thereby effectively mitigating any inherent intra-run variability. The ultimate assay output was reported in picograms per milliliter (pg/mL), predicated upon a robust 4-parameter logistic (4-Pl) fit model, thereby ensuring precise absolute quantification. Comprehensive insights into the assay’s validation parameters, encompassing limits of detection, intra- and inter-assay precision data, and related metrics, are available at https://olink.com

Output from the Olink software was further processed to extrapolate values for samples that were below the LLOQ and above the upper limit of quantification (“>ULOQ”). The Olink software interpolates values below the LLOQ through fitting the 4-Pl model to a distinct minimum limit of detection for each plate of samples run, and values below this interpolation range are set to NaN. Since these values are below the limit of detection but not truly missing, for each assay, we determined a universal “below detection” value by taking the mode LLOQ across 22 plates, divided by 10,000 (which was below all interpolated values in this extensive historical dataset), and set all NaN to this assay-specific below detection value. Samples above the ULOQ were set to the ULOQ value for the indicated target assay from the plate on which the sample was run. Finally, true missing values annotated as “No Data” were converted to NA to be systematically treated as missing.

### Statistics.

Statistical analysis was performed using the Mann-Whitney test to compare continuous factors between 2 groups or generalized estimating equations (as implemented with glmgee in the geepack R package) to assess changes within animals of each group over time. Correlation analyses were performed using the 2-tailed nonparametric Spearman rank correlation method. All statistical tests were performed as 2-tailed tests. Since our research design was as hypothesis-generating, exploratory research, all *P* values are reported as nominal values without adjusting for multiple comparisons. *P* < 0.05 was considered statistically significant.

### Study approval.

The NIH NCI Animal Care and Use Committee, Bethesda, Maryland, USA, approved the vaccine study. Animals were housed and maintained at the NCI animal facility at the NIH, Bethesda, Maryland, USA. The NIH is accredited by Association for Assessment and Accreditation of Laboratory Animals International and follows the Public Health Service Policy for the Care and Use of Laboratory Animals. Animal care was provided in accordance with the procedures outlined in the *Guide for Care and Use of Laboratory Animals* (National Research Council; 1996; National Academies Press). All animal care and procedures were carried out under protocols approved by the NCI and/or NIAID Animal Care and Use Committee before study initiation. Animals were closely monitored daily for any signs of illness, and appropriate medical care was provided as needed. Animals were socially housed per the approved Animal Care and Use Committee protocol and social compatibility. All clinical procedures, including biopsy collection, administration of anesthetics and analgesics, and euthanasia, were carried out under the direction of a laboratory animal veterinarian. Steps were taken to ensure the welfare of the animals and minimize discomfort of all animals used in this study. Animals were fed daily with a fresh diet of primate biscuits, fruit, forage, and other food items to maintain body weight or normal growth. Animals were monitored for mental health and provided with physical enrichment, including sanitized toys, destructible enrichment (cardboard and other paper products), and audio and visual stimulation.

### Data availability.

Data and code used to generate all the figures in the manuscript can be found at Zenodo at https://zenodo.org/records/15845110 The Supporting Data Values file contains values plotted for each panel of each figure.

## Author contributions

MAR, TC, and GF conceived the study. MAR, KCG, and GF wrote the manuscript, with contributions from all authors. MAR coordinated the macaque studies; handled SARS-CoV-2–infected samples, phenotyping, and intracellular cytokine assays in BAL, blood, and mucosal tissues; analyzed the data; and wrote the first draft. KCG analyzed all data and prepared most figures. SS performed VL assay, handled SARS-CoV-2–infected samples, kept inventory, and maintained the samples. MB performed efferocytosis assay and T cell phenotyping, and handled SARS-CoV-2–infected samples;.AG performed VL assay and helped with different in vitro assays. L Schifanella performed Olink assay. RM and MND processed samples and performed experiments. HA performed plaque reduction neutralization test assay. YJ and L Serebryannyy performed antibody assay. MGL coordinated the SARS-CoV-2 infection in the animals.

## Supplementary Material

Supplemental data

Supporting data values

## Version 1 08/08/2025

Electronic publication
