# Supplementary material for: BCG immunization mitigates SARS-CoV-2 replication in macaques via monocyte efferocytosis and neutrophil recruitment in lungs
Source: JCI Insight. 2025 Aug 8;10(15):e194633. doi: 10.1172/jci.insight.194633 (PMC12333941; doi:10.1172/jci.insight.194633)
Supplement: Supplemental data [file jciinsight-10-194633-s008.pdf]

Supplemental Material

JCI Insight

**Article Title:**	**BCG immunization mitigates SARS-CoV-2 replication in macaques via monocyte efferocytosis and neutrophil recruitment in lung**
**Corresponding Author:**	Mohammad Arif Rahman Ph.D. mohammadarif.rahman@nih.gov<br>Genoveffa Franchini, franchig@mail.nih.gov

Supplemental Item & Number	Title or Caption
Supplemental Figure 1	Input virus viral load (VL) and replicating sgRNA VL in different respiratory tract compartments
Supplemental Figure 2	Representative flow cytometry gating strategy of neutrophils and macrophages/monocytes
Supplemental Figure 3	Association between replicating viral load (VL) in bronchoalveolar lavage (BAL) at day 7 and vaccine-induced immune parameters
Supplemental Figure 4	Correlations between immune parameters, replicating viral load at day 7 in bronchoalveolar lavage (BAL), and efferocytosis in peripheral blood
Supplemental Figure 5	Association of immune parameters with replicating viral load in bronchoalveolar lavage (BAL) at Day 7
Supplemental Figure 6	T cell gating strategy
Supplemental Figure 7	Binding antibody responses against spike and receptor-binding domain (RBD) protein
Supplemental Figure 8	Summary of immune responses associated with reduced replicating SARS-CoV-2 in bronchoalveolar lavage (BAL)
Supplemental Figure 9	Summary of immune responses associated with increased replicating SARS-CoV-2 in bronchoalveolar lavage (BAL)
Supplemental Table 1	Plasma neutralizing antibodies to SARS-CoV-2

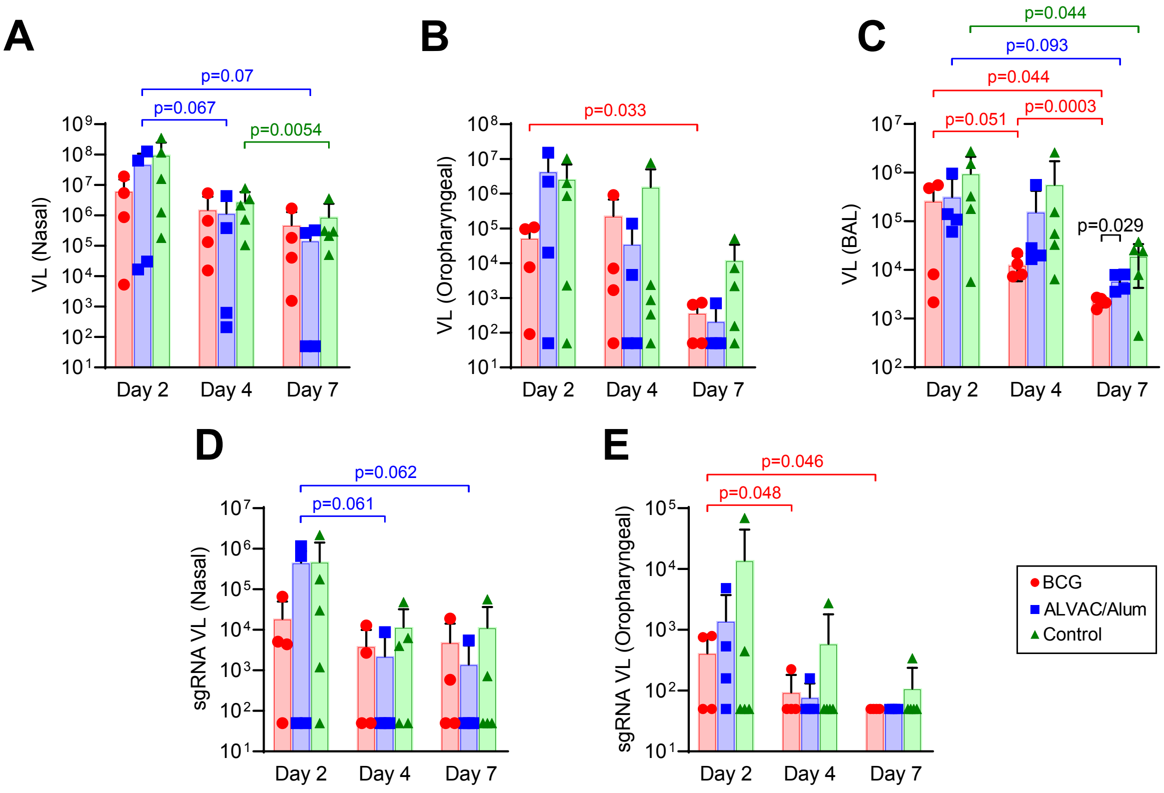

F

Group or Timepoint	Comparison	VL Nasal p_raw	VL Nasal p_adj	VL Oropharengeal p_raw	VL Oropharengeal p_adj	VL BAL p_raw	VL BAL p_adj	repVL Nasal p_raw	repVL Nasal p_adj	repVL Oropharengeal p_raw	repVL Oropharengeal p_adj	repVL BAL p_raw	repVL BAL p_adj
BCG	Day4vsDay2	0.24	0.24	0.34	0.34	0.051	0.051	0.31	0.53	0.048	0.072	0.038	0.053
BCG	Day7vsDay2	0.13	0.24	0.033	0.099	0.044	0.051	0.37	0.53	0.046	0.072	0.039	0.053
BCG	Day7vsDay4	0.16	0.24	0.24	0.34	0.00025	0.00075	0.53	0.53	0.25	0.25	0.053	0.053
ALVAC	Day4vsDay2	0.067	0.105	0.17	0.23	0.52	0.52	0.061	0.093	0.18	0.25	0.15	0.15
ALVAC	Day7vsDay2	0.07	0.105	0.17	0.23	0.093	0.279	0.062	0.093	0.18	0.25	0.13	0.15
ALVAC	Day7vsDay4	0.23	0.23	0.23	0.23	0.2	0.3	0.25	0.25	0.25	0.25	0.15	0.15
Control	Day4vsDay2	0.12	0.12	0.68	0.68	0.48	0.48	0.22	0.345	0.26	0.33	0.18	0.25
Control	Day7vsDay2	0.11	0.12	0.13	0.39	0.044	0.132	0.23	0.345	0.26	0.33	0.11	0.25
Control	Day7vsDay4	0.0054	0.0162	0.27	0.405	0.23	0.345	0.99	0.99	0.33	0.33	0.25	0.25
Day2	BCGvsALVAC	0.69	0.69	0.69	0.81	0.69	0.69	0.88	0.9	0.88	0.89	0.69	0.73
Day4	BCGvsALVAC	0.49	0.49	0.66	0.9	0.11	0.285	0.62	0.7	1	1	0.46	0.69
Day7	BCGvsALVAC	0.66	0.66	0.62	0.62	0.029	0.087	0.62	1	n/v	n/v	0.026	0.078
Day2	BCGvsControl	0.41	0.69	0.56	0.81	0.56	0.69	0.62	0.9	0.89	0.89	0.56	0.73
Day4	BCGvsControl	0.41	0.49	0.81	0.9	0.19	0.285	0.7	0.7	1	1	0.26	0.69
Day7	BCGvsControl	0.29	0.585	0.26	0.39	0.19	0.285	1	1	0.5	0.5	0.073	0.109
Day2	ALVACvsControl	0.56	0.69	0.81	0.81	0.41	0.69	0.9	0.9	0.61	0.89	0.73	0.73
Day4	ALVACvsControl	0.29	0.49	0.9	0.9	0.56	0.56	0.5	0.7	1	1	0.81	0.81
Day7	ALVACvsControl	0.39	0.585	0.12	0.36	0.29	0.29	0.77	1	0.5	0.5	0.41	0.41

Supplemental Figure 1. Input virus viral load (VL) and replicating sgRNA VL in different respiratory tract compartments. (**A, B, C**) VL was determined in (**A**) nasal swab, (**B**) oropharyngeal swab, and (**C**) bronchoalveolar lavage (BAL) post SARS-CoV-2 viral challenge. sgRNA replicating VL was determined in (**D**) nasal swab and (**E**) oropharyngeal swab post SARS-CoV-2 viral challenge. (**F**) summary p-value table with raw and adjusted p values for each category/comparison/analyte. p-values that are labeled in the plots are also colored in this table and calculated by fitting generalized estimating equations (between timepoints for each group separately) or two-tailed Mann-Whitney test (between groups at each timepoint). Barplots denote mean and error bars are SD.

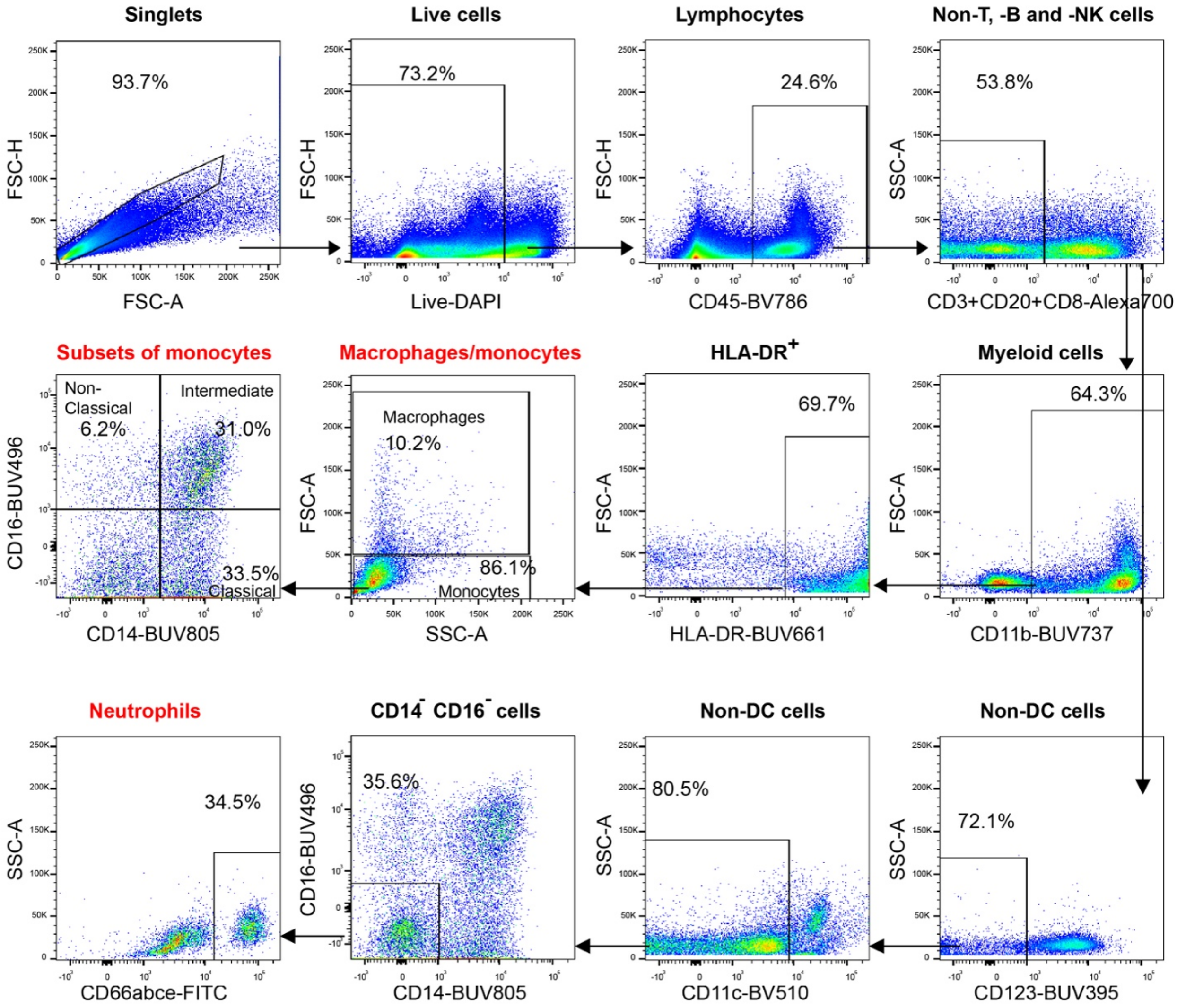

Supplemental Figure 2. Representative Flow Cytometry Gating Strategy for Identification of Neutrophils and Macrophages/Monocytes. The figure illustrates the sequential gating strategy employed to identify neutrophils and macrophages/monocytes in peripheral blood and bronchoalveolar lavage (BAL) samples.

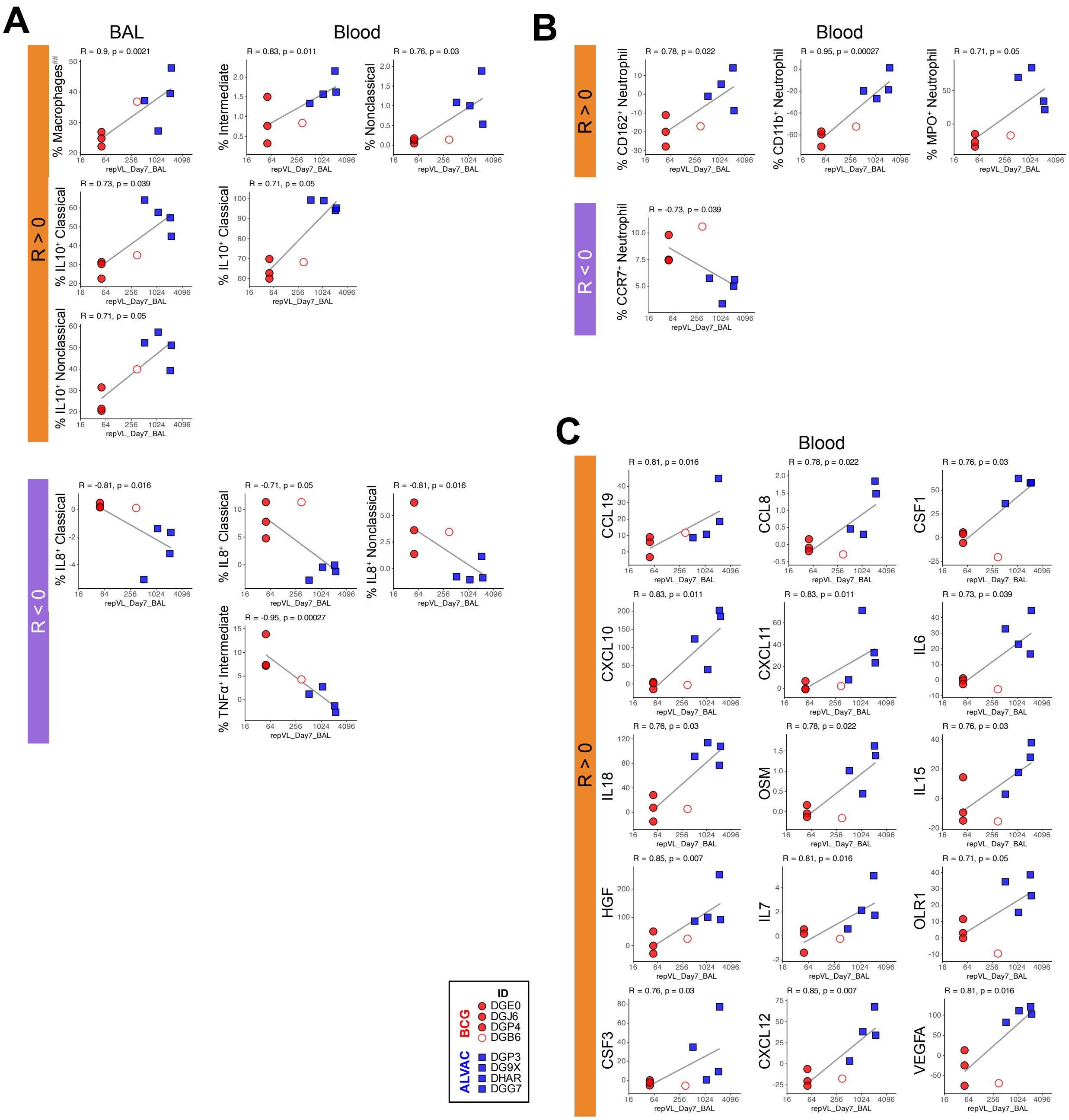

Supplemental Figure 3. Association between replicating viral load (VL) in bronchoalveolar lavage (BAL) at day 7 and vaccine-induced immune parameters. (A–C) Correlation analyses were performed between replicating VL in BAL at day 7 post-challenge and vaccine-induced immune responses, calculated as the change from pre-vaccination baseline to 2 days post-vaccination for each parameter. Data points represent individual animals; corresponding Spearman correlation coefficients (R) and two-tailed p-values are indicated in each panel. Only analytes with two-tailed Spearman $p < 0.05$ are shown. Hollow circle designates BCG-vaccinated animal that did not control replicating VL.

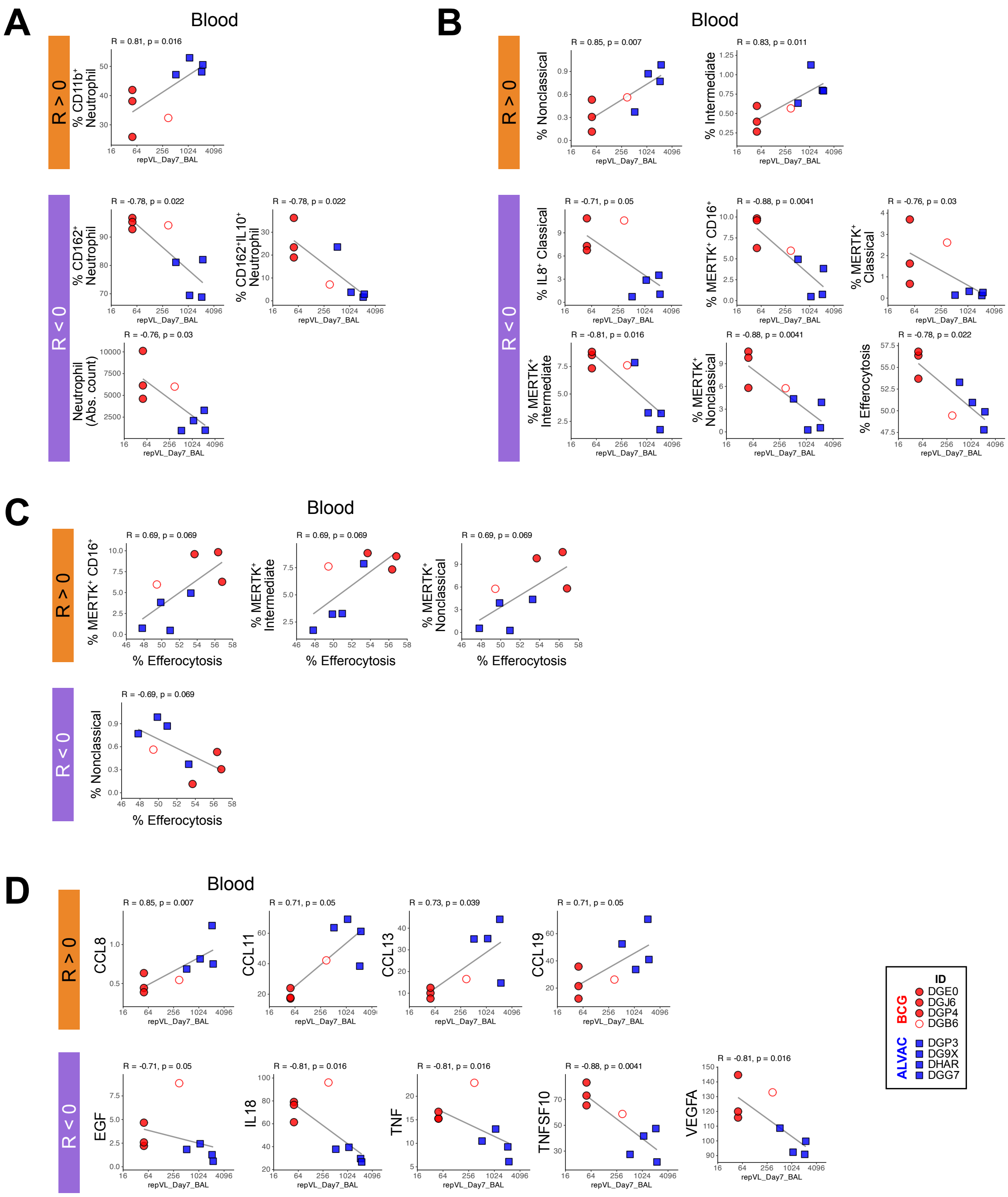

Supplemental Figure 4. Correlations between immune parameters, replicating viral load at day 7 in bronchoalveolar lavage (BAL), and efferocytosis in peripheral blood. (A, B, D) Correlation of selected immune parameters with replicating viral load (VL) in BAL at day 7 post-infection. **(C)** Correlation of immune parameters with levels of efferocytosis in blood at pre-SARS-CoV-2 infection (Pre-S). Data points represent individual animals; corresponding Spearman correlation coefficients (R) and two-tailed p-values are indicated in each panel. Only analytes with two-tailed Spearman $p < 0.05$ are shown. Hollow circle designates BCG-vaccinated animal that did not control replicating VL.

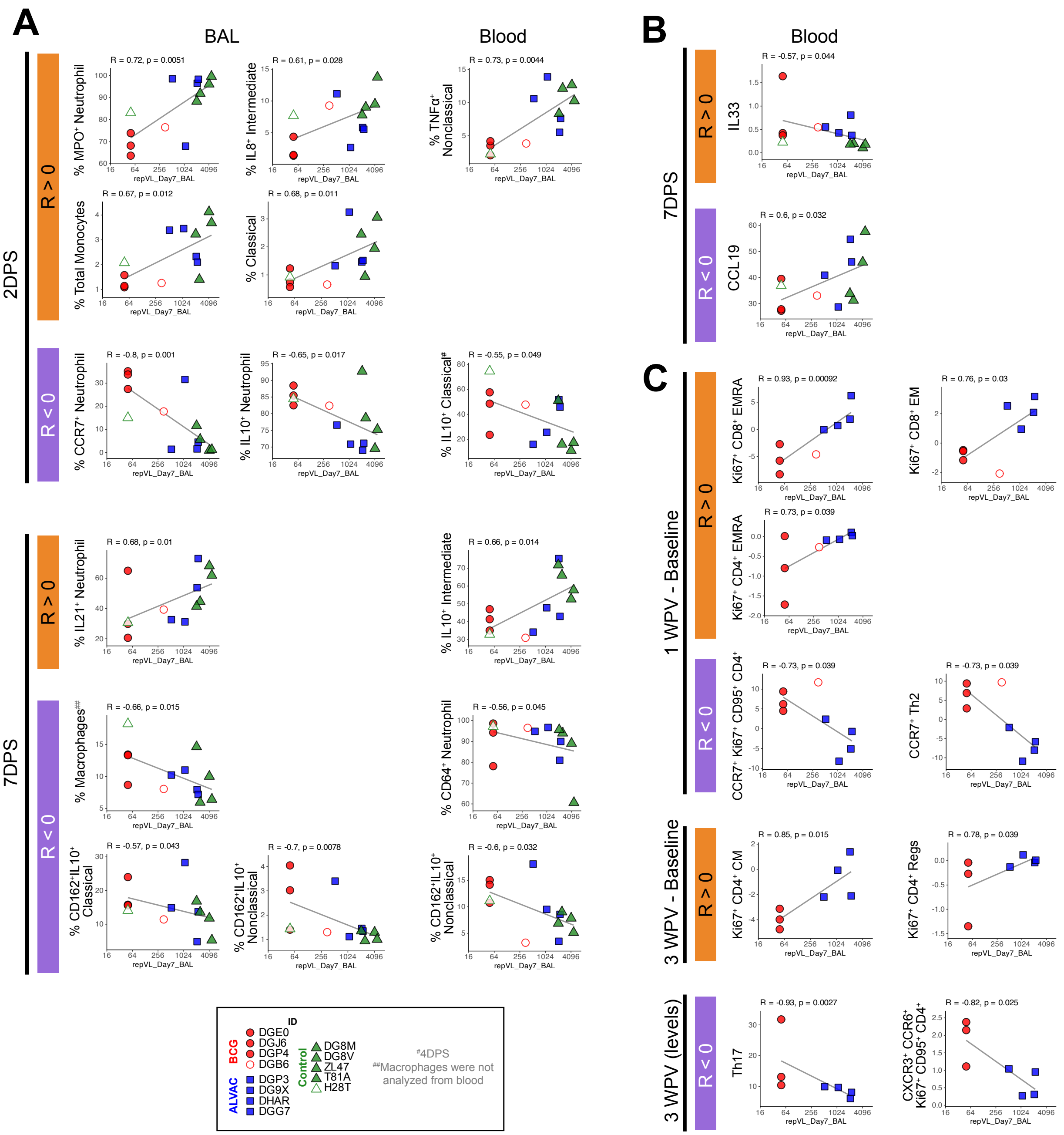

Supplemental Figure 5. Association of Immune Parameters with Replicating Viral Load (VL) in bronchoalveolar lavage (BAL) at Day 7. (A–C) Association between selected immune parameters and replicating VL in BAL samples at day 7 post-infection. Data points represent individual animals; corresponding Spearman correlation coefficients (R) and two-tailed p-values are indicated in each panel. Only analytes with two-tailed Spearman $p < 0.05$ are shown. Hollow circle designates BCG-vaccinated animal that did not control replicating VL, hollow triangle highlights the nonvaccinated animal that controlled replicating VL.

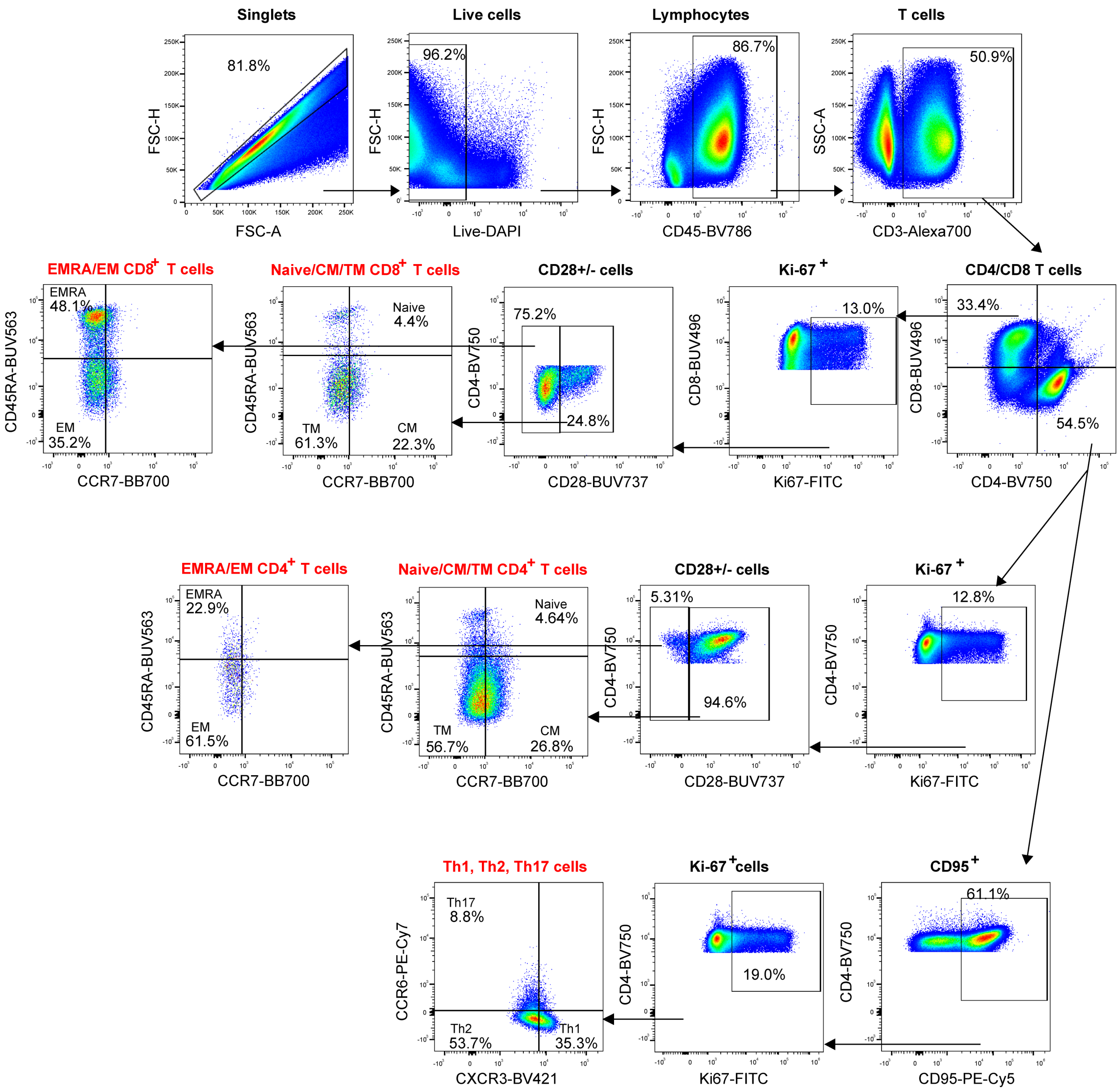

Supplemental Figure 6. T Cell Gating Strategy in blood. Representative flow cytometry gating strategy used to identify $CD4^+$/ $CD8^+$ T cell subsets of naïve T cells, central memory (CM), effector memory (EM), transitional memory (TM), RA^+ effector memory (EMRA), as well as Th1, Th2, Th17 cells.

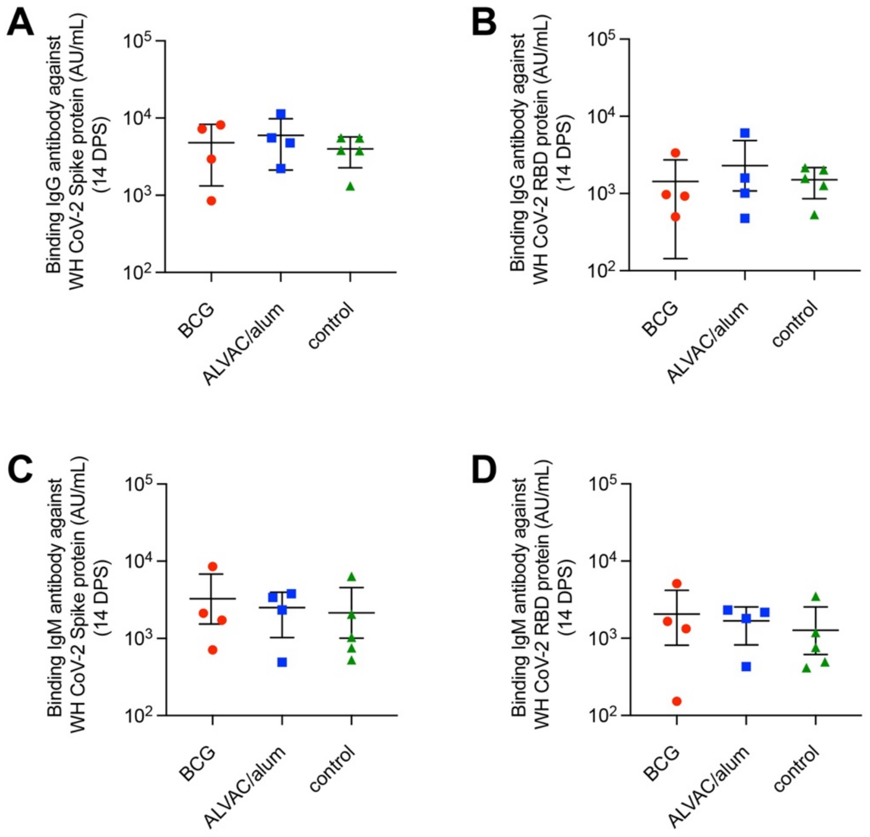

Supplemental Figure 7. Binding antibody responses against spike and receptor-binding domain (RBD) protein in plasma. Comparison of (**A**) IgG binding against spike, (**B**) IgG binding against RBD, (**C**) IgM binding against spike, and (**D**) IgM binding against RBD among different groups of animals at 14 days post SARS-CoV-2. Data shown in (**A-D**) were analyzed with two-tailed Mann-Whitney U test. Horizontal and vertical bars denote mean and SD.

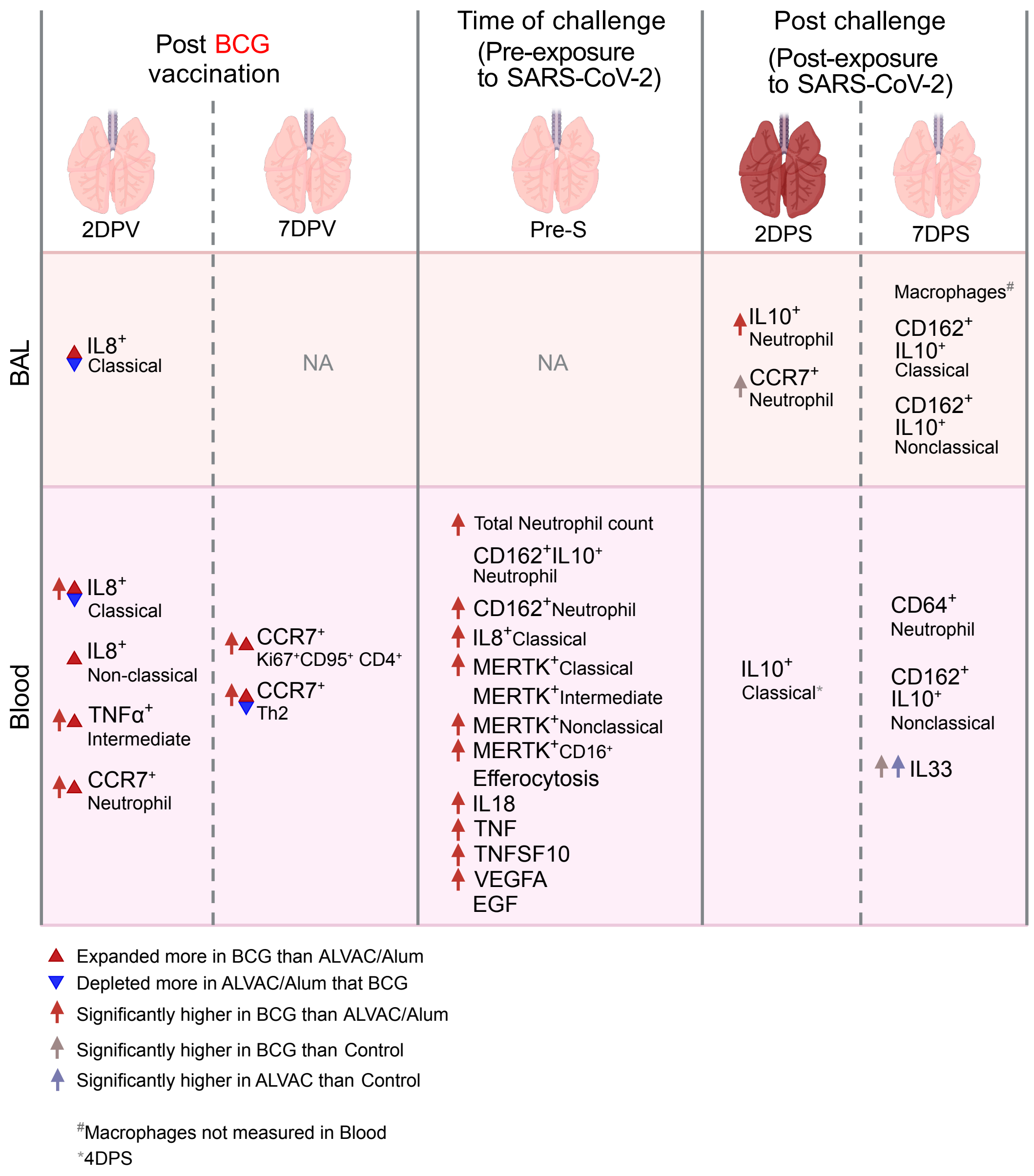

Supplemental Figure 8. Summary of immune responses associated with reduced replicating SARS-CoV-2 in bronchoalveolar lavage (BAL). Cell populations and cytokines associated with reduced replicating SARS-CoV-2 are summarized by time (left to right) and compartment (top to bottom). Triangles indicate analytes expanded more over time in BCG than ALVAC/Alum (up, red) or depleted more over time in ALVAC/Alum than BCG (down, blue). Arrows indicate analyte levels that are higher in BCG vs ALVAC/Alum (red), BCG vs non-vaccinated control (muted red), or ALVAC/Alum vs non-vaccinated control (muted blue).

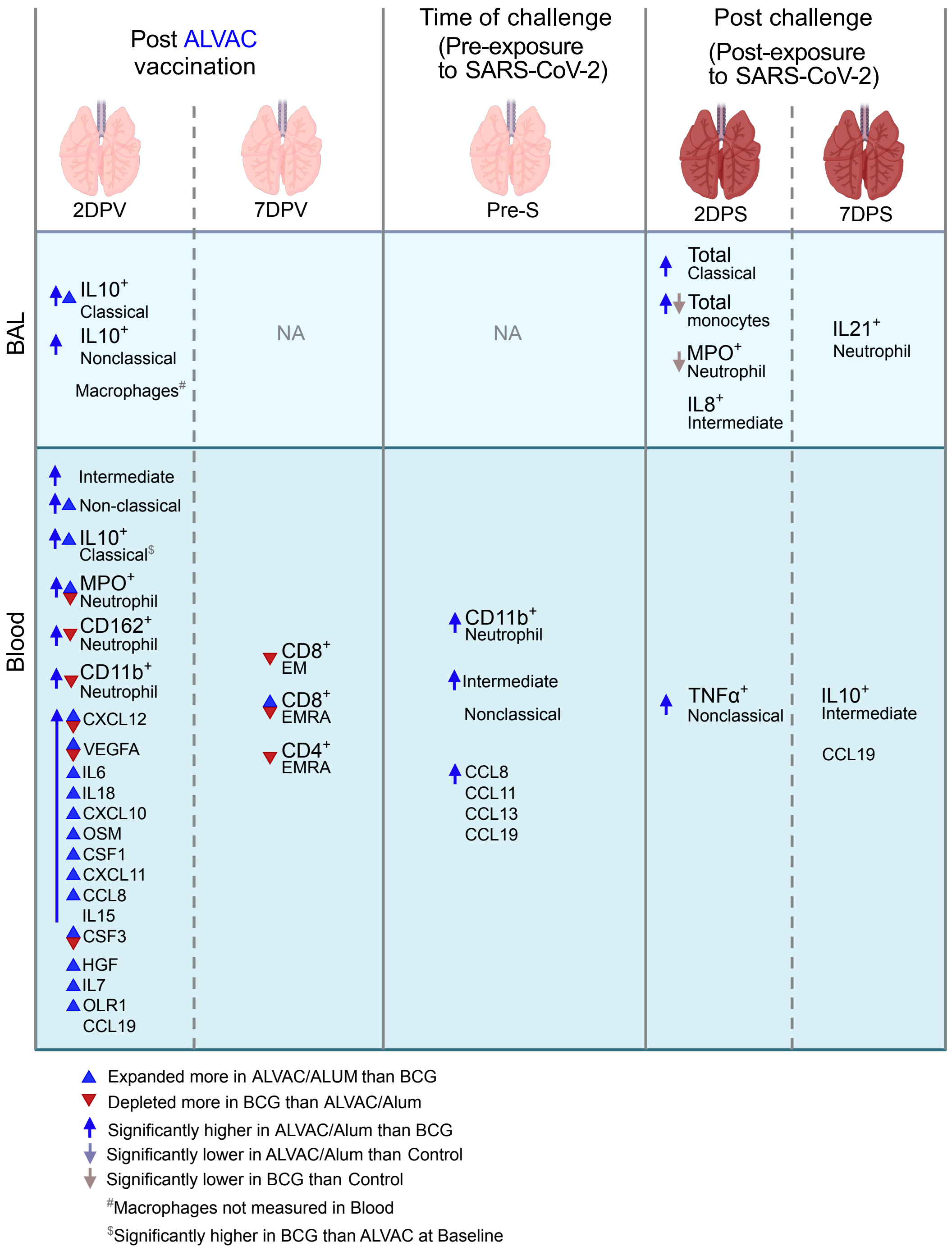

Supplemental Figure 9. Summary of immune responses associated with increased replicating SARS-CoV-2 in bronchoalveolar lavage (BAL). Cell populations and cytokines associated with increased replicating SARS-CoV-2 are summarized by time (left to right) and compartment (top to bottom). Triangles indicate analytes expanded more over time in ALVAC/Alum than BCG (up, blue) or depleted more over time in BCG than ALVAC/Alum (down, red). Arrows indicate analyte levels that are higher in ALVAC/Alum vs BCG (up, blue), or BCG vs non-vaccinated control (down, muted red).

	Animal ID	Experimental Control	Pre	7 days post vaccine	Pre-exposure	7 days post exposure	14 days post exposure
BCG	DGE0		<20	<20	<20	<20	<20
BCG	DGJ6		<20	<20	<20	<20	<20
BCG	DGB6		<20	<20	<20	<20	<20
BCG	DGP4		<20	<20	<20	<20	<20
ALVAC/Alum	DGP3		<20	<20	<20	<20	<20
ALVAC/Alum	DHAR		<20	<20	<20	<20	<20
ALVAC/Alum	DG9X		<20	<20	<20	<20	<20
ALVAC/Alum	DGG7		<20	<20	<20	<20	<20
Naïve control	DG8M				<20	<20	<20
Naïve control	DG8V				<20	<20	<20
Naïve control	T81A				<20	<20	<20
Naïve control	ZL47				<20	<20	<20
Naïve control	H28T				<20	<20	<20
Positive Control	Rabbit reference serum	1,620					

Supplemental Table 1. Plasma neutralizing antibodies to SARS-CoV-2. Neutralization assay was performed against 2019-nCoV/USA-WA1/2020 SARS-CoV-2 strain in plasma samples collected at baseline (Pre), 7 days following last vaccination, 14 days prior (Pre-exposure), and 7- and 14-days following SARS-CoV-2 exposure.
